# A revision of the bioregionalisation of freshwater fish communities in the Australian Monsoonal Tropics

**DOI:** 10.1002/ece3.5059

**Published:** 2019-03-29

**Authors:** James J. Shelley, Tim Dempster, Matthew C. Le Feuvre, Peter J. Unmack, Shawn W. Laffan, Stephen E. Swearer

**Affiliations:** ^1^ School of BioSciences University of Melbourne Melbourne Victoria Australia; ^2^ Institute for Applied Ecology University of Canberra Canberra Australian Capital Territory Australia; ^3^ School of Biological Earth and Environmental Sciences University of New South Wales Sydney New South Wales Australia

**Keywords:** aquatic communities, biogeography, environmental drivers, northern Australia, species turnover *β*_sim_, tropics

## Abstract

The Australian freshwater fish fauna is very unique, but poorly understood. In the Australian Monsoonal Tropics (AMT) biome of northern Australia, the number of described and candidate species has nearly doubled since the last attempt to analyse freshwater fish species composition patterns and determine a bioregionalisation scheme. Here, we utilise the most complete database of catchment‐scale freshwater fish distributions from the AMT to date to: (a) reanalyze spatial patterns of species richness, endemism and turnover of freshwater fishes; (b) propose a biogeographic regionalisation based on species turnover; (c) assess the relationship between species turnover and patterns of environmental change and historic drainage connectivity; and (d) identify sampling gaps. Biogeographic provinces were identified using an agglomerative cluster analysis of a Simpson's beta (*β*_sim_) dissimilarity matrix. A generalised dissimilarity model incorporating eighteen environmental variables was used to investigate the environmental correlates of species turnover. Observed and estimated species richness and endemism were calculated and inventory completeness was estimated based on the ratio of observed to estimated species richness. Three major freshwater fish biogeographic provinces and 14 subprovinces are proposed. These differ substantially from the current bioregionalisation scheme. Species turnover was most strongly influenced by environmental variables that are interpreted to reflect changes in terrain (catchment relief and confinement), geology and climate (runoff perenniality, stream density), and biotic responses to climate (net primary productivity). Past connectivity between rivers during low sea‐level events is also influential highlighting the importance of historical processes in explaining contemporary patterns of biodiversity in the AMT. The inclusion of 49 newly discovered species and candidate species only reinforced known focal points of species richness and endemism in the AMT. However, a number of key sampling gaps remain that need to be filled to fully characterise the proposed bioregionalisation.

## 1 INTRODUCTION

**Figure 1:**
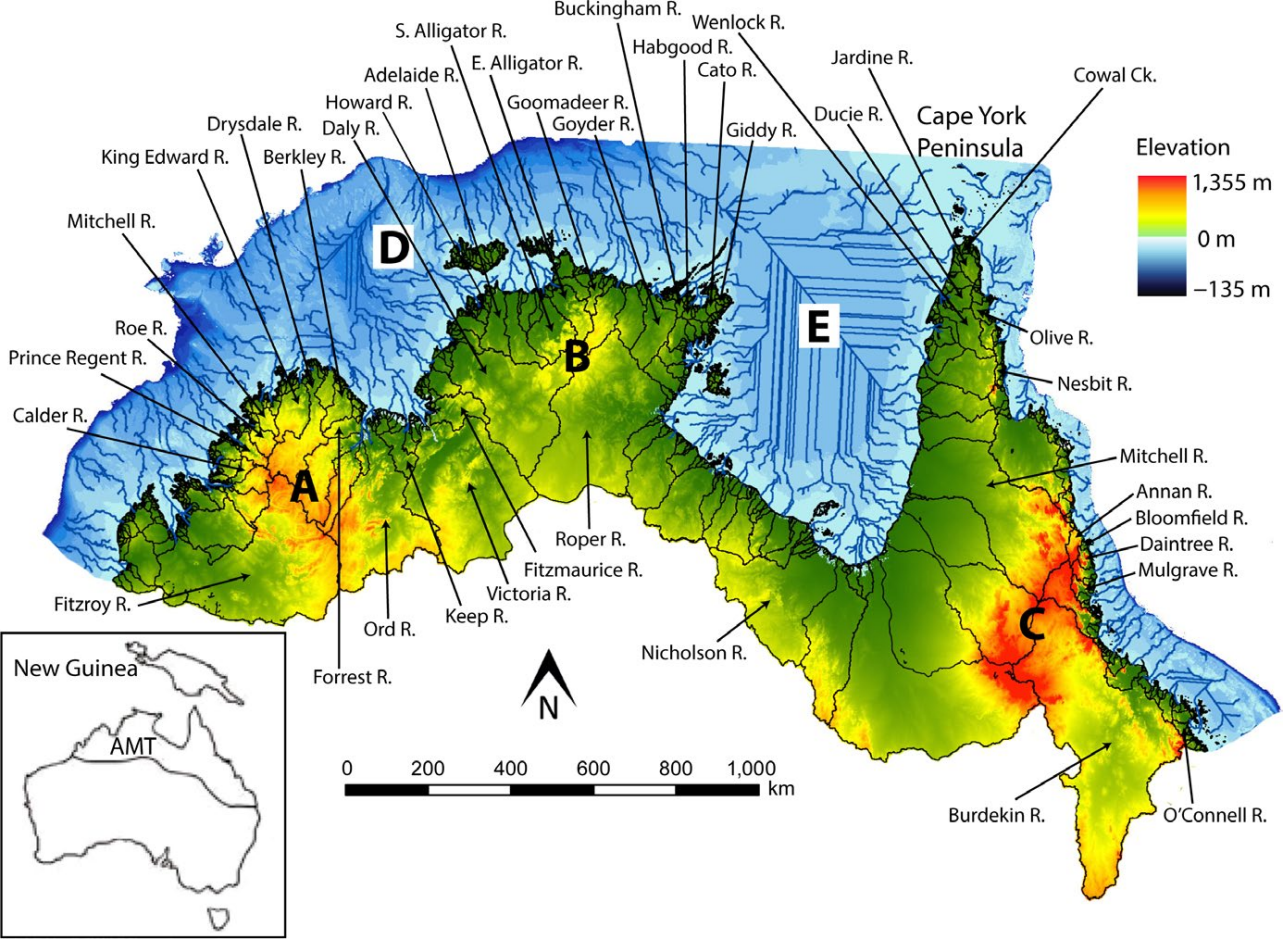
Elevation map of the Australian Monsoonal Tropics and catchment boundaries. Arrows indicate catchments discussed in text. Letters denote prominent geographic features discussed in text: (a) Kimberley Plateau, (b) Arnhem Plateau, (c) Great Dividing Range, (d) Lake Bonaparte, (e) Lake Carpentaria. Beyond the coastline, the sea floor depth to 135 m (the maximum estimated fall in sea level during the last glacial cycle; Clark & Mix, 2002) is marked by a gradient of blue. The margin of the figure represents the 135 m below sea‐level contour and effectively highlights the continental shelf edge. Low sea‐level drainage patterns are shown as blue lines, derived from a bathymetric 30 arc‐second (c. l km) dataset from the Australian Geological Survey Organization

In comparison to other continents, Australian freshwater fish communities are considered highly unique but species poor, largely due to Australia's arid climate and long isolation from other land masses (Unmack, 2001, 2013). However, the Australian Monsoonal Tropics (AMT) biome in the tropical north of the continent is an exception (see Figure 1). The AMT encompasses 33% of Australia's landmass, but contains 65% of all freshwater fishes, and the encompassed provinces contain a moderate number of species compared to others around the globe (Abell et al., 2008). Furthermore, the number of described and candidate species in the AMT has increased by 50% since the turn of the millennium, compared to a background rate of 35% across all of Australia (see Allen, Midgley, & Allen, 2002; Le Feuvre, Dempster, Shelley, & Swearer, 2016 for comparison). Despite its importance and the disproportionate increase in biodiversity estimates, the AMT has been the subject of considerably less research into biodiversity and the evolution of biotic communities than the highly populated regions to the south (Bowman et al., 2010). There is therefore a substantial need for increased biogeographic research in this region.

The most recent analysis of freshwater fish distribution patterns (excluding diadromous species; Unmack, 2001) mapped species richness, endemism, and general biogeographic provinces at the continental scale. The analysis was constrained to a comparison of relatedness between operational geographic units (OGUs) defined by expert opinion. This work was updated by Unmack (2013) accounting for newly discovered species, but the bioregionalisation was not reanalyzed. The substantial increase in biodiversity estimates and changes recorded in species distributions in the AMT is likely to challenge aspects of the original analysis.

Furthermore, accurate and comprehensive models of landscape scale environmental data are now readily available and can be used to quantitatively explore potential drivers of freshwater fish species distributions in the AMT (e.g., Hermoso, Kennard, & Linke, 2012; Sternberg & Kennard, 2013; Sternberg & Kennard, 2014). Our understanding of the historical significance of sea‐level changes, driven by Plio‐ Pleistocene glacial cycles, on species distributions has also become clearer since phylogenetic data collection and analysis have become more widely implemented. There is an increasing amount of phylogenetic evidence that the particularly wide continental shelf around the northern coastline of the AMT provided dispersal pathways for a number of freshwater species between regions that are now isolated, for example between the island of New Guinea and Australia (see Figure 1; e.g., Baker, De Bruyn, & Mather, 2008; Cook, Adams, Mather, & Hughes, 2012; De Bruyn, Wilson, & Mather, 2004; Huey, Cook, Unmack, & Hughes, 2014). A key remaining challenge is to untangle the influence of past and present processes on biogeographic patterns. All of these elements combined highlight the need and opportunity to produce a comprehensive and updated analysis of freshwater fish biogeography in the AMT.

By grouping biological communities into clusters of meaningful geographical units, patterns in evolution, diversity, and the ecological processes that sustain that diversity can be revealed (Morrone & Crisci, 1995). As such, a biogeographic regionalisation is essential for developing ecologically representative systems of protected areas and is a requirement of national and international conservation agreements such as the Ramsar Convention on Wetlands, the National Water Initiative, and the Australian Guidelines for Establishing the National Reserve System (Kennard, 2010; Lourie & Vincent, 2004; Olden et al., 2010). It is also an important tool to assess our current state of knowledge, identify survey gaps to prioritise future research, and to refine biogeographic province boundaries (Higgins, Bryer, Khoury, & Fitzhugh, 2005).

Here, we generate a bioregionalisation of the freshwater fishes in the Australian tropics using the Simpson's beta dissimilarity metric, and then assess the relationships of biogeographic regions, referred to here as “provinces” and “subprovinces”, to their current environment using generalised dissimilarity modeling (GDM). Specifically, our goals were to: (a) map spatial patterns of species richness, endemism and turnover between river catchments; (b) propose an updated biogeographic regionalisation of freshwater fishes based on the spatial turnover of species; (c) assess the relationship between species turnover and patterns of environmental change and historic drainage connectivity; and (d) identify survey gaps that require further sampling.

## 2 METHODS

We investigated biogeographic patterns of freshwater fishes in the AMT, revising the most recent analysis conducted by Unmack (2001). We did not attempt to exactly replicate the analysis in Unmack (2001) as new techniques have become available and are better suited to this dataset (see *Geographical structure of species turnover*). Key differences between these two studies are outlined in Table 1.

**Table 1:** Principal differences between the present study and that of Unmack (2001) for the analysis of the distribution of species dissimilarity and diversity of freshwater fishes

Study parameters	Unmack 2001	Present study
Study area	Australia	AMT
Number of species (AMT)	93 (diadromous species excluded)	178 (diadromous species included)
Dissimilarity measure	Dice, Ochiai, Kulczynski, Jaccard	Beta‐Simpson index
Cluster metric	UPGMA, NMDS, parsimony	WPGMA
Environmental variables considered	3 (elevation, rainfall, temperature)	18 (see Table 2)
Environmental statistics	None	GDM
Spatial units	Operational geographic units of varying sizes	Catchment
Terminology	Province, subprovince, and region (comparable to our subprovinces)	Province and subprovince
Diversity metrics used	Species richness and absolute endemism	Species richness and corrected weighted endemism
Number of bioregions	3 provinces, 3 subprovinces, 14 regions	3 provinces, 14 subprovinces

### 2.1 Study area

The AMT biome is here defined as encompassing all coastal river catchments from the Fitzroy River in the Kimberley region eastwards to the O'Connell River on Australia's east coast (Figure 1). As a biome, the southern limit is defined by areas that receive more than 85% of their annual rainfall between November and April, commonly referred to as the “wet season” (Bowman et al., 2010). South of the southern border of the AMT lies the vast arid and semi‐arid expanses of inland Australia. The 200 mm/year rainfall contour marks a hard faunal break for aquatic communities. The break is less distinct across catchments that drain to the east coast where rainfall is significantly higher. A strong faunal connection exists between the AMT and southern New Guinea with the regions sharing 66 species (37% of the total). The AMT landscape is largely flat, and geologically stable, with the most recent major tectonic activity (e.g., mountain building) occurring >80 Ma (Johnson, 2004). Concentrated human development is limited across the region and the river catchments represent some of the most intact riverine ecosystems in the world (Pusey et al., 2011). However, Indigenous Australians have occupied Australia for approximately 50,000 years (Bowler et al., 2003; Flood, 2004) and their impact on the distribution of freshwater fishes is poorly understood (Humphries, 2007). All things considered, the AMT presents an ideal region in which to study natural freshwater biogeographic patterns.

### 2.2 Species distribution dataset

#### 2.2.1 Published species distributions

Our definition of freshwater fish includes species that reproduce in freshwater and diadromous species that spend part of their life history in freshwater. Species distributions for each catchment were primarily derived from three comprehensive and relatively recent reviews of fish distributions across the study region. These include reviews of the Kimberley region, northwestern Australia, from the Fitzroy River to the Ord River (Morgan, Allen, Pusey, & Burrows, 2011) and from the Fitzroy River to the Fitzmaurice River (Shelley, Morgan et al., 2018), northern Australia from the Keep River (immediately east of the Ord River) to the Annan River in Queensland (Pusey et al., 2017), and between the Annan River and the O'Connell River at the southern extent of our study area (Pusey, Kennard, & Arthington, 2004). Readers can refer to these papers for complete reference lists of the surveys that contributed to the underlying data. Additional data was added to these lists from more recent surveys, which are listed in Supporting Information Appendix S1. Anomalous records were crosschecked with authorities on freshwater fish distributions in northern Australia (see Acknowledgments).

#### 2.2.2 Inclusion of genetic candidate species

Taxonomy is fluid in that names of species, genera and families change and new “cryptic species” are discovered within previously described groups as new data become available. There are complexes of species awaiting formal description in Australia (and elsewhere) that show genetic structure that likely warrants taxonomic recognition (Unmack, 2013). As molecular techniques become more affordable, the identification of cryptic species within widespread groups is becoming increasingly common. Unfortunately, taxonomy is a poorly funded science in Australia and taxonomists are unable to keep pace with morphological evaluation and description of the ever‐increasing backlog of genetically defined candidate species.

This taxonomic situation is unlikely to be resolved in the near future and as such we believe that, in the interest of conservation of these candidate species, they should be included in conservation frameworks as we are attempting to define here. While morphological analysis may or may not support these genetic distinctions, strictly morphological based descriptions are not infallible either and are also subject to change. With this in mind, we included 32 genetic candidate species from the study region that await formal morphological examination (Unmack, 2013). These included likely species complexes listed in Humphries and Walker (2013), compiled by Michael Hammer and Peter Unmack and those identified in Shelley, Morgan et al. (2018) and Shelley, Swearer et al. (2018). A list of the candidate species, the morphological and genetic evidence from which their candidate species status was determined, and the references for that evidence is provided in Supporting Information Appendix S2. Furthermore, in line with Pusey et al. (2017), three species groups ((a) *Zenarchopterus buffonis*,* Z. caudovittatus* and *Z. novaeguineae*; (b) *Ophisternon bengalense* and *O. guttarale*; and (c) *Leptachirus polylepis* and *L. darwinensis*) were collapsed into generic groups (*Zenarchopterus* spp., *Ophisternon* spp. and *Leptachirus* spp.) as their morphological similarity has cast considerable doubt over the accuracy of individual species records in Northern Australia.

#### 2.2.3 Final database

River catchments were used as the base spatial unit for our analyses and included any sized creek or river catchment defined in the river catchment framework from the Australian Hydrologic Geospatial Fabric (Stein, Hutchinson, & Stein, 2014), which was accessed using ArcGIS 10.1 (ESRI, 2012). The river catchment framework is derived from a 9 arc‐second digital elevation model (DEM) stream network (ANU Fenner School of Environment and Society and Geoscience Australia, 2008). Based on a visual assessment of the frequency distribution of catchment species richness across the AMT, we identified catchments with fewer than 10 species records as potentially data deficient outliers. We then used expert opinion to determine whether these outliers were likely to be naturally species poor or whether the low species count was an artifact of low sampling effort. Three catchments (Berkley [Western Australia], King, and West Alligator [Northern Territory]) were considered data deficient and were not included in our analysis. Our final database included 178 species of described and candidate freshwater bony fishes known from 112 catchments across the study region (Supporting Information Appendix S3).

### 2.3 Environmental datasets

A range of ecologically relevant catchment‐scale environmental variables were selected from a larger number of candidate variables for use in GDM. Environmental data for each catchment unit were derived from the National Environmental Stream Attributes database (Stein et al., 2014) that was linked to the catchments defined in the Australian Hydrologic Geospatial Fabric Surface Network layer, using ArcGIS. The candidate variables described climate, terrain, substrate, natural vegetation cover, hydrology, stream network characteristics, and terrestrial primary productivity. Principal Component Analysis and Spearman's correlations among candidate variables were used to identify and remove those that were highly correlated. The final subset of predictor variables used in our analysis is listed in Table 2. Spearman's correlation coefficients among the final sets of predictor variables ranged between −0.5 and +0.6.

**Table 2:** Environmental and distance variables used in our analyses

Environmental/distance variable	Description
Net primary productivity (tC/ha^13^)	Annual mean net primary productivity (terrestrial)
Interannual variation in annual runoff	Coefficient of variation of annual totals of accumulated soil water surplus
Annual mean runoff (ML)	Mean of the annual totals of the monthly accumulated soil water surplus values
Perenniality of runoff (%)	Percentage contribution to mean annual discharge by the six driest months of the year
Annual mean rainfall (mm)	Annual mean rainfall of all grid cells within the catchment
Annual mean temperature (°C)	The average of all weekly maximum and minimum temperatures (°C) within the catchment
Annual mean radiation (MJ m^−2^ day^−1^)	The mean of all the weekly radiation estimates within the catchment
Stream density (km/km^2^)	Total length of all stream segments in the stream network/contributing catchment area
Wateriness (%)	Proportion of the catchment that contains waterholes or springs or is occupied by a lake or the watercourse
Catchment area (km^2^)	Area contributing to the catchment
Maximum upstream elevation (m)	Highest catchment elevation value
Catchment relief (ratio)	Mean upstream elevation of each stream segment/maximum upstream elevation
Catchment average slope (%)	The average slope for each stream segment within the catchment
Stream confinement (%)	Percentage of stream segments that flow through open plains rather than confined valleys
Mean segment elevation (m)	Mean elevation of each individual stream segment
Vegetation bare (%)	Stream and valley percentage naturally bare
Vegetation natural grasses (%)	Stream and valley percentage natural grasses cover
Vegetation natural forests (%)	Stream and valley percentage natural forests cover
Current geographical distance (no. of catchments)	Number of catchment divides that have to be crossed between any two given catchments along the coastline
Paleo‐geographical distance (no. of catchments)	Number of catchment divides that have to be crossed between any two given paleo‐drainages along the coastline at the −135 m contour

*Notes*

Environmental variables were derived from the National Environmental Stream Attributes database (Stein, Hutchinson, & Stein, 2014). Calculations for the “Paleo‐geographical distance” were based on paleo‐drainage models derived from a bathymetric 30 arc‐second (c. l km) dataset produced by the Australian Geological Survey Organization (see Figure 1). Modelled paleo‐drainage connections at the sea floor depth of −135 m (the maximum estimated fall in sea level during the last glacial cycle; Clark & Mix, 2002) were used.

### 2.4 Paleo‐drainages

Drainage patterns during lowered sea levels (paleo‐drainages) were modelled using Spatial Analyst 1.1 and ArcView 3.1, based upon a bathymetric 30 arc‐second grid produced by the Australian Geological Survey Organization. Paleo‐drainages are displayed from the current coastline to the sea floor depth of −135 m (the maximum estimated fall in sea level during the last glacial maximum; Clark & Mix, 2002).

### 2.5 Current and paleo‐geographic distance between drainage basins

To account for a decrease in the species compositional similarity between two localities with increasing geographical distance (i.e., distance‐decay; Nekola & White, 1999), we calculated measures of geographical distance between (a) current catchments as defined in the river catchment framework from the Australian Hydrologic Geospatial Fabric (Stein et al., 2014); and (b) paleo‐catchments defined in the modeled low sea‐level drainage patterns (see Figure 1). Similar to the methodology of Dias et al. (2014), we defined the current geographic distance between river catchments as the number of catchment divides that would have to be crossed along the coastline when travelling from one basin to another. While some catchments may border one another at their headwaters, freshwater fish dispersal via headwater capture events are considered to be extremely rare in the geologically stable AMT (Unmack, 2001), hence we focused on dispersal across catchment divides at the coastline. In this way, we accounted for dispersal constraints on species movement, and thus species turnover, in addition to ecological niche‐based processes that are accounted for by the environmental variables in the model, described above. This approach was also preferable as it overcomes the difficulty of defining the geographical Euclidean distances between river catchments.

To calculate current geographical distance, catchments (including those without fish species composition data) between the western and eastern‐most rivers used in our analysis were numbered in increasing order (i.e., from the Fitzroy River [1] to the O'Connell River [301]). In this way, a distance of 1 corresponds to catchments that are adjoining at the coast, while that number increases the further apart they are. The corresponding catchment number was ascribed to each catchment for which we had fish community data.

To investigate the effect of low sea‐level catchment connectivity on species turnover, we calculated paleo‐geographic distances using the same approach as described above to the paleo‐catchments at the −135 m contour. For example, all catchments that drained into the paleo‐lake, Lake Carpentaria, during the lowered sea levels are labeled a single number. We exclusively considered the confluence of drainages at the lowest sea level reached during the last glacial maximum (−135 m) as it presents the greatest possible opportunity for connectivity between catchments.

Spearman's correlation analysis was used to test how highly correlated current and paleo‐geographical distances were. The resulting Spearman's correlation coefficient (0.81) indicated that the two variables are quite highly correlated, highlighting the difficulty in untangling the influences of past and present connectivity on species turnover. In response to this, we ran the GDMs with and without paleo‐geographical distance and found that its inclusion improved the amount of variance accounted for in the whole AMT model by 8.5%, and the province scale models by as much as 11.8% (results not presented here). Given the significant improvement in explanatory power of the models, we included paleo‐geographical distance as a variable in our analysis.

### 2.6 Species richness and endemism

We calculated species richness and endemism to identify spatial patterns of ecological and conservation significance. Many diversity patterns are scale dependent, in that some occur over geographically local extents, while others become apparent only when much larger spatial extents are considered (Laffan & Crisp, 2003). Here, we analyze endemism at three biologically relevant spatial scales defined by our analysis of species turnover (see section below): biogeographic province, subprovince, and catchment.

Species richness (SR) and corrected weighted endemism (CWE; Crisp, Laffan, Linder, & Monro, 2001) were calculated in Biodiverse 1.99 (Laffan, Lubarsky, & Rosauer, 2010) using catchments as the finest spatial unit. We considered endemism in relation to the AMT; that is, species distributed outside the study region may still be considered endemic to a given catchment, subprovince, or province within the AMT. CWE is a relative measure of endemism that indicates the degree of range restriction of a sample to a location or set of locations. In the case of this study, it can be interpreted as the degree to which species found in a spatial unit (e.g., catchment or subprovince), on average, are restricted to that unit (Laffan, Ramp, & Roger, 2013). Once the species richness and endemism scores were calculated for all catchments, we defined focal points of species richness and endemism by selecting those catchments with the highest 10% of scores.

### 2.7 Species turnover

A matrix of Simpson's beta (*β*_sim_) species turnover was generated for all pairwise catchment combinations for use in our cluster analysis. Simpson's beta was used because it reduces the effect of any species richness imbalance between locations and thus reduces the effect of unequal sampling effort (Tuomisto, 2010), which is characteristic of this poorly studied region. Simpson's beta can be calculated as

$$\beta_{{\mathrm{sim}}_{i,j}}=1-\frac{a}{a+\text{min}(b,c)}$$

where *a* refers to the number of species common to spatial units *i* and *j*,* b* is the number found in spatial unit *i* but not spatial unit *j*, and *c* is the number found in spatial unit *j* but not spatial unit *i*. A low *β*_sim_ value indicates that many taxa are shared between two spatial units (low dissimilarity) and a high *β*_sim_ means a small number of shared taxa (high dissimilarity).

### 2.8 Geographical structure of species turnover

The *β*_sim_ pairwise distance matrix was used in an agglomerative cluster analysis to generate a WPGMA (weighted pair‐group method using arithmetic averages) hierarchical dendrogram in Biodiverse. This allowed us to visually assess the relationships between the catchments (i.e., the similarity of their species composition) and ultimately determine a bioregionalisation scheme. WPGMA weights the contributions of clusters by the number of terminal nodes (spatial units) they contain, ensuring each unit contributes equally to each merger of which it is a part. Kreft and Jetz (2010) found WPGMA was consistently among the best performing hierarchical clustering methods. We used a tiebreaker approach such that, when multiple pairs of clusters had the minimum turnover score and thus could be merged, the algorithm selected the pair that maximised the corrected weighted endemism score in the cluster (González‐Orozco et al., 2014; Laffan & Crisp, 2003). Using this approach provides a more stable and replicable result while also optimising for the degree of endemism and thus spatial compactness of the resultant biogeographic regions. Our criteria for defining biogeographic regions (provinces and subprovinces) from the clusters were: (a) respective regions are represented by a contiguous group of catchments; and (b) each cluster that represents a region is clearly separated from its children or parent in the dendrogram. Furthermore, we set a limit where only the first 10% of nodes (from the root of the dendrogram) represented divergences that were deep enough to be classed as a subprovince.

### 2.9 Generalised dissimilarity modeling

We used GDM to examine how freshwater fish species composition varies with climate, geography and habitat, using the Generalised Dissimilarity Modeler 1.2.3. GDM models dissimilarity in species composition between pairs of locations as a nonlinear function of geographical and environmental distances between these locations. Unlike other linear approaches, GDM allows for curvilinear relationships between observed compositional dissimilarity and increasing ecological and geographical separation between sites, and variation in the rate of compositional turnover at different positions along environmental gradients (for a full description of the method see Ferrier, Manion, Elith, & Richardson, 2007). The analysis was run using a Simpson's beta dissimilarity matrix, the subset of uncorrelated environmental predictors, and geographical distance between the centroids of sampled catchments. We ran the analysis for the biome (AMT) and individual provinces, to help determine the most significant environmental drivers at each level. A Wald test was applied to each parameter to determine if it significantly impacted model fit (*p*‐value ≤0.05).

### 2.10 Inventory completeness index

To identify poorly sampled regions that are most in need of additional sampling effort, we calculated an inventory completeness index (C‐index). The C‐index can be defined as the ratio of observed species richness to estimated species richness in a given spatial unit (Soberón, Jiménez, Golubov, & Koleff, 2007).

We calculated estimates of potential species richness using the nonparametric Chao 2 estimator (Colwell & Coddington, 1994) implemented in Biodiverse. The Chao 2 estimator calculates the total number of species present, including those species that were not sampled, by extrapolating the asymptote of a rarefaction curve. For a given spatial unit *i* (e.g., province) this statistic (*S*_chao(*i*)_) can be calculated as

$$S_{\mathrm{chao}(i)}=S_{\mathrm{obs}(i)}+\left(f_{1}^{2}/2f_{2}\right)$$

where *S*_obs(*i*)_ is the observed species richness in spatial unit *i*, and *f*_1_ and *f*_2_ are the number of singletons (species represented in a single sampling unit) and doubletons (species represented in two sampling units), respectively, found in *i*. The completeness index (C_index_) was then calculated as

$$C_{\mathrm{index}}=S_{\mathrm{obs}(i)}/S_{\mathrm{chao}(i)}$$

We analyzed inventory completeness at the three conservation management spatial levels (biome, province, and subprovince). Due to its formulation, the Chao 2 index cannot be accurately estimated for a single spatial unit such as a catchment, so catchment level estimates were not included.

## 3 RESULTS

### 3.1 Biogeographic provinces

**Figure 2:**
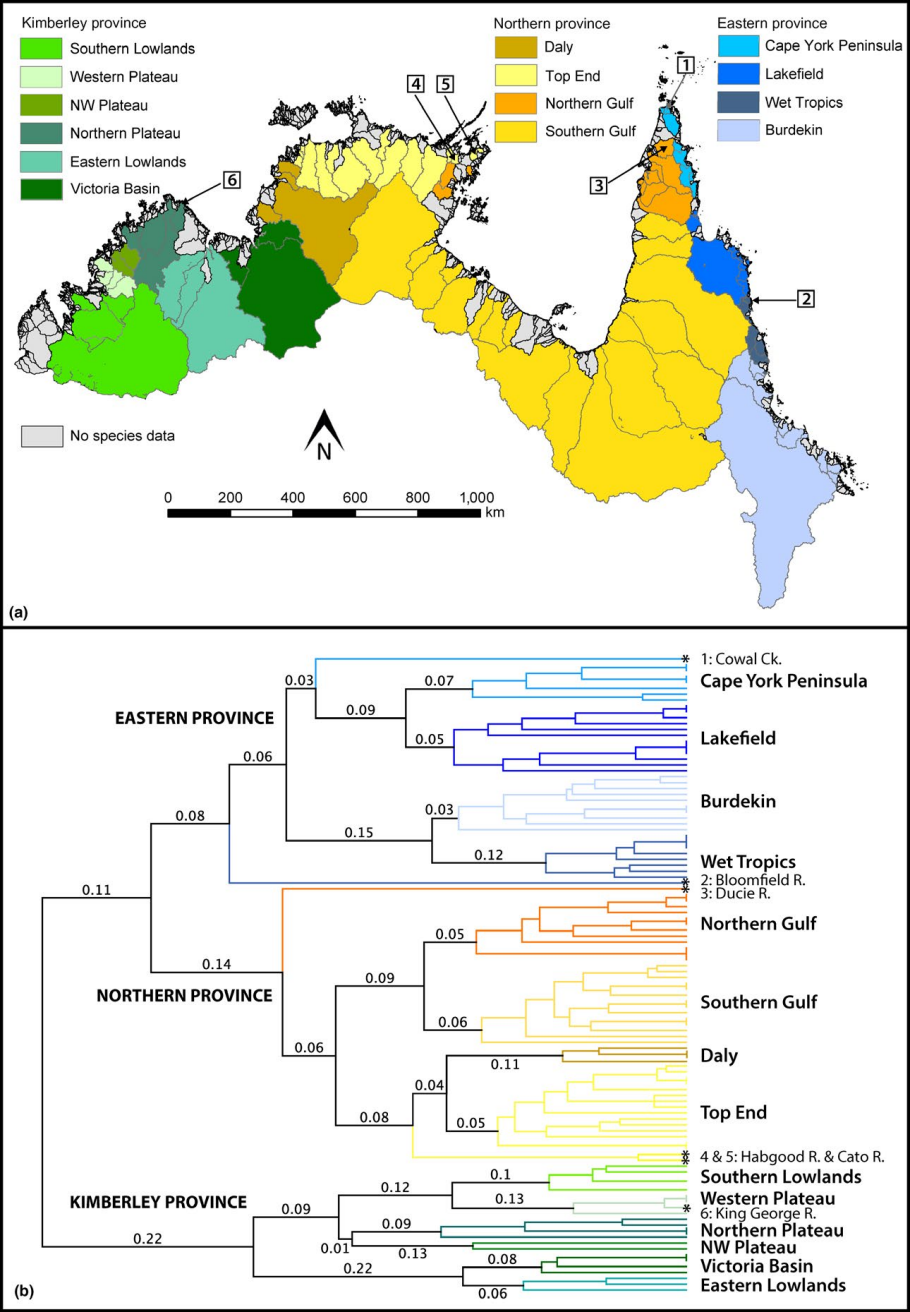
Proposed freshwater fish biogeographic provinces (in capitals) and subprovinces (in lower case) in the Australian Monsoonal Tropics (a) and the WPGMA tree of 178 species based on Simpson's beta dissimilarity matrix upon which the provinces were determined (b). Numbered boxes in (a) refer to anomalous catchments highlighted in (b) and are discussed in the Results section. Asterisks at the end of branches in (b) denote anomalous catchments. Gray catchments in (a) are those for which no or inadequate fish community composition data was available

Three major biogeographic provinces and 12 subprovinces are proposed within the AMT biome (Figure 2). The dendrogram branch lengths (Table 3) for each of the geographical clusters (Figure 2b) show 8%–22% divergence (species‐composition dissimilarity) between the three major provinces (Kimberley, Northern, and Eastern), with the Kimberley being by far the most divergent region. Kimberley Province is further subdivided into five subprovinces that show 6%–13% divergence from their nearest sister cluster. Southern Lowlands are most similar to the neighboring Western Plateau subprovince, the NW Plateau is most similar to the Northern Plateau, and the Eastern Lowlands form an independent cluster with the Victoria Basin. While the King George River clustered with the Western Plateau rivers, we designate it herein as being part of Northern Plateau as it is situated adjacent to the rivers in that subprovince. The anomalous positioning of the King George River is likely due to its depauperate fish community (seven species) of which six are widespread species. A steep 80 m waterfall is present on the mainstream near the coastline, which appears to prevent dispersal of most species from neighboring catchments. Its positioning may change as the phylogeographic affinities of its populations are revealed and further sampling is conducted in this particularly remote corner of the Kimberley Plateau.

**Table 3:** Values of branch length, number of catchments surveyed (*n*), corrected weighted endemism (CWE), observed species richness (*S*_obs_), incidence‐based estimate of species richness ± *SE* (*S*_est_), and estimated completeness of the inventory (range; C_index_), based on *S*_est_ for clusters of freshwater fish provinces and subprovinces in the Australian Monsoonal Tropics

Provinces (bold) and subprovinces	Branch length	*n*	CWE	*S*_obs_	*S*_est_	C_index_
Australian Monsoonal Tropics		109		179	199.42 ± 6.43	0.90 (0.87–0.93)
**Kimberley**	**0.22**	**20**	**0.64**	**78**	**92.03 ± 8.69**	**0.85 (0.77–0.94)**
Southern Lowlands	0.13	4	0.29	29	33.49 ± 2.80	0.87 (0.80–0.94)
Western Plateau	0.10	4	0.27	22	23.34 ± 1.74	0.94 (0.88–1.00)
NW Plateau	0.13	2	0.35	31	36.16 ± 3.65	0.86 (0.78–0.95)
Northern Plateau	0.09	5	0.30	37	46.55 ± 7.36	0.79 (0.69–0.94)
Eastern Lowlands	0.06	3	0.19	38	43.04 ± 4.18	0.88 (0.80–0.98)
Victoria Basin	0.08	3	0.12	41	43.29 ± 2.07	0.95 (0.90–0.99)
**Northern**	**0.16**	**52**	**0.68**	**90**	**98.36 ± 1.36**	**0.93 (0.91–0.94)**
Daly	0.17	5	0.17	49	51.72 ± 3.14	0.95 (0.89–1.00)
Top End	0.05	16	0.38	62	60.38 ± 2.14	1.00 (0.99–1.00)
Northern Gulf	0.08	13	0.19	60	64.21 ± 3.25	0.93 (0.89–0.98)
Southern Gulf	0.12	16	0.35	56	73.59 ± 8.57	0.77 (0.69–0.88)
**Eastern**	**0.08**	**39**	**0.68**	**97**	**99.17 ± 2.30**	**0.98 (0.96–1.00)**
Cape York Peninsula	0.07	8	0.16	56	60.00 ± 4.65	0.93 (0.87–1.00)
Lakefield	0.05	12	0.21	49	53.49 ± 4.48	0.92 (0.85–1.00)
Wet Tropics	0.12	9	0.39	66	67.36 ± 2.28	0.98 (0.95–1.00)
Burdekin	0.02	10	0.31	52	54.84 ± 2.01	0.97 (0.93–1.00)

The Northern Province comprises four large subprovinces that exhibit 5%–17% divergence from their nearest sister cluster. Daly is most closely related to Top End while Northern Gulf is most closely related to Southern Gulf. Northern Gulf encompasses catchments on either side of the Gulf of Carpentaria and, given that these catchments would have been connected under low sea‐level conditions (Figure 1), they were considered to be geographically contiguous. Three anomalous catchments were apparent within the Northern Province that were most strongly defined by an absence of many common species, rather than the presence of unique species. The Habgood and Cato rivers formed an independent cluster most closely related to Daly and Top End, but we designate them herein as being part of Top End as (a) they were most closely related to the main Top End cluster (9%); and (b) these rivers are geographically situated between the Buckingham and Giddy rivers that are within the main Top End cluster. In the case of the Habgood and Cato rivers, their anomalous positioning in the cluster is likely due to the comparatively low sampling effort they have received. Furthermore, the Ducie River was found to be independent of all Northern subprovinces, but we designate it as being part of Northern Province and Northern Gulf subprovince herein as (a) it was most closely related to Northern Gulf (20%); (b) the river geographically lies adjacent to the other rivers within the Northern Gulf cluster; and (c) it marks the distributional limit for three widespread Northern Province species (*Scortum ogilbyi*,* Brachirus salinarum* and *B. selheimi*) and is just beyond the distributional limit of three widespread Eastern Province species (*Melanotaenia maccullochi* I, *Anguilla obscura* and *A. reinhardtii*).

Eastern Province is divided into four subprovinces separated from their nearest sister cluster by 3%–12%. Cape York Peninsula and Lakefield are most similar and form a cluster that is independent of the more closely related Wet Tropics and Burdekin subprovinces. Two anomalous catchments were apparent within the Northern Province (Cowal Creek and Bloomfield River) that again were most strongly defined by an absence of common species, rather than the presence of unique species. However, the Bloomfield River is also home to a narrow‐range endemic species, the Bloomfield River Cod (*Guyu wujalwujalensis*). Cowal Creek was found to be similar to, but independent of, Cape York Peninsula and Lakefield. We designate it as being part of Cape York Peninsula as (a) although it is slightly more closely related to Lakefield, it contains five species that are found in other Cape York Peninsula streams (*Ambassis elongata*,* Iriatherina werneri* I, *Melanotaenia nigrans*,* Neosilurus brevidorsalis*,* Oxyeleotris fimbriata*) but not in Lakefield streams, while it shares no species with Lakefield that are not in other Wet Tropics streams; and (b) the river lies adjacent to the other rivers within the Wet Tropics cluster. The Bloomfield River was found to be independent of all Eastern subprovinces, but we designate it as part of the Wet Tropics as (a) although it is slightly more closely related to the other Eastern subprovinces, it contains nine species that are otherwise endemic to Wet Tropics (*Sicyopterus lagocephalus*,* Si. discordipinnis*,* Smilosicyopus fehlmanni*,* Stiphodon atratus*,* St. alleni*,* St. birdsong*,* St. rutilaureus*,* St. semoni* and *Synclidopus hogani*) but shares no endemic species from the other subregions; and (b) the river lies adjacent to the other rivers within the Wet Tropics cluster.

### 3.2 Species richness and endemism

**Figure 3:**
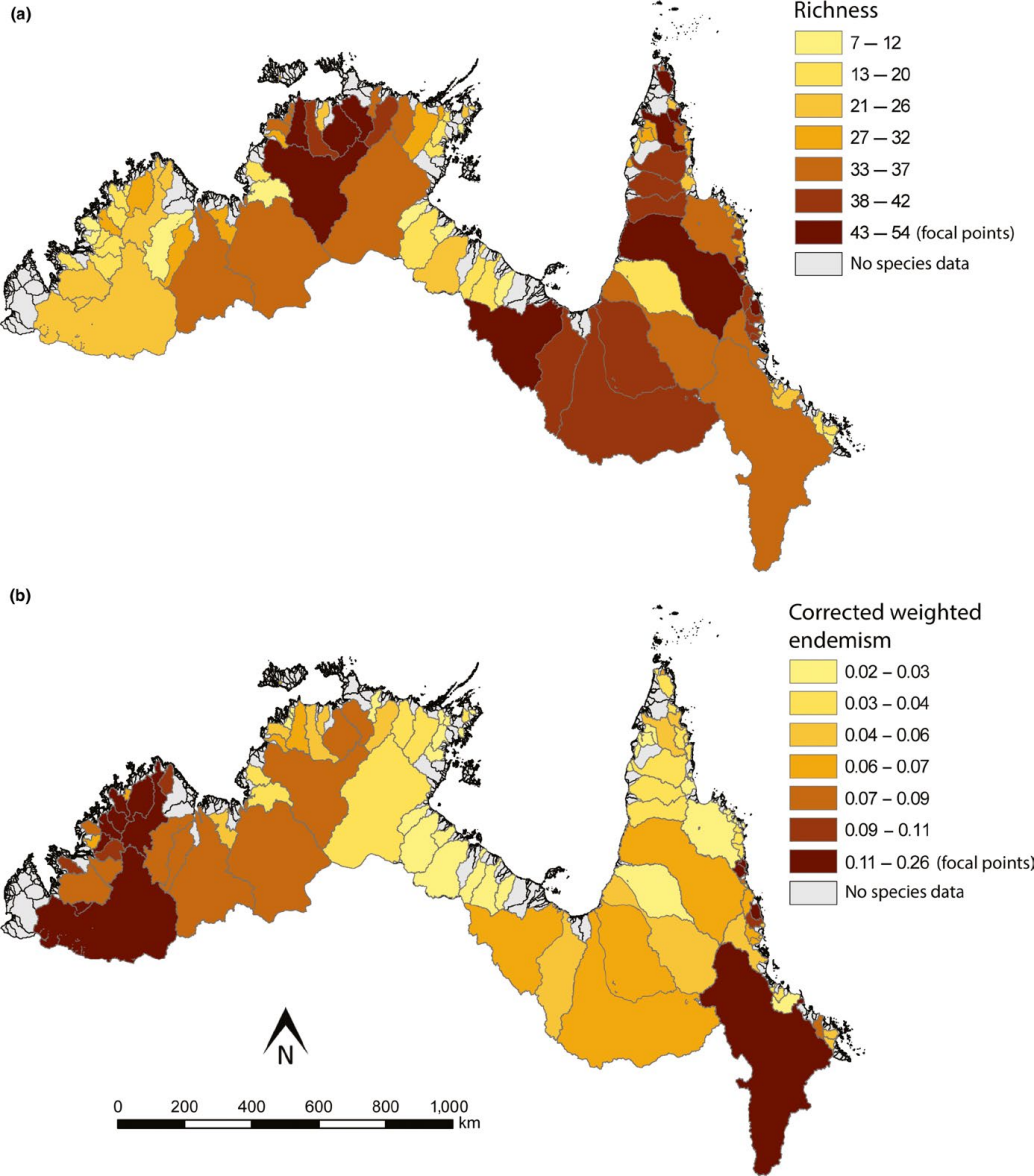
Per‐catchment values of (a) species richness, and (b) corrected weighted endemism in the Australian Monsoonal Tropics. Gray catchments are those for which no or inadequate fish community composition data was available

The most species rich and endemic regions of freshwater fishes showed little overlap (Figure 3, Table 3). At the catchment level, focal points of species richness (43–54 species per catchment) were spread across the Northern and Eastern Provinces, specifically the Daly (Daly River), Top End (Adelaide, East Alligator, South Alligator rivers), Northern Gulf (Wenlock rivers), and Southern Gulf (Nicholson and Mitchell rivers) subprovinces of the Northern Province, and the Cape York Peninsula (Jardine and Olive River) and Wet Tropics (Daintree and Mulgrave rivers) subprovinces of Eastern Province (Figure 3a). None were identified in the Kimberley. In total, Wet Tropics was the most species rich subprovince (66 species) and Eastern was the most species rich province (97 species).

While species richness was moderate, Kimberley Province was identified as a focal point of narrow‐range endemism (CWE 0.11–0.26), specifically in the Northern Plateau (Mitchell, King Edward and Drysdale rivers), NW Plateau (Prince Regent and Roe rivers), Western Plateau (Calder River), and Southern Lowlands (Fitzroy River; Figure 3b, Table 3). The Bloomfield, Daintree and Mulgrave rivers in the Wet Tropics and the Burdekin River in Burdekin were also focal points of narrow‐range endemism. At the subprovince level, Wet Tropics had the highest endemism score (CWE = 0.39), while at the province level Northern and Eastern shared the highest score (CWE = 0.68).

### 3.3 Generalised dissimilarity modeling

**Figure 4:**
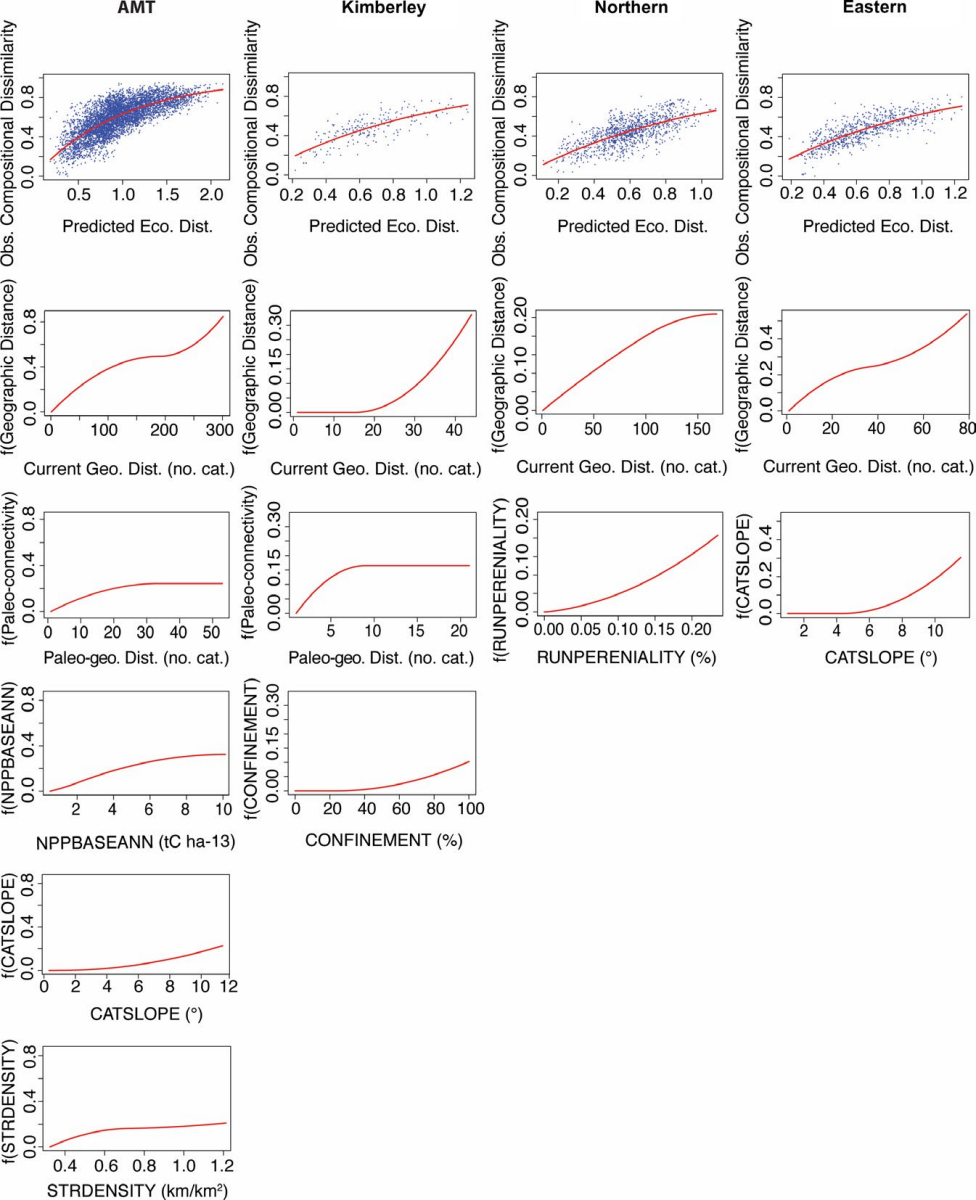
Predicted ecological distance of the generalised dissimilarity model of freshwater fish species composition in the Australian Monsoonal Tropics and each major province plotted against observed compositional dissimilarity (top row), and functions fitted to each of the most significant environmental and distance predictors (*p*‐value ≤0.05) in the generalised dissimilarity model of freshwater fish species composition. The figure is arranged in columns by biome/province. Abbreviations for significant predictors relate to Table 2 descriptions as follows: Current Geo. Dist. = Current geographic distance, Paleo‐geo. Dist. = Paleo‐geographic distance, NPPBASEANN = Net primary productivity, CONFINEMENT = Stream confinement, CATSLOPE = Catchment average slope, STRDENSITY = Stream density, RUNPERENIALITY = Perenniality of runoff

**Figure 5:**
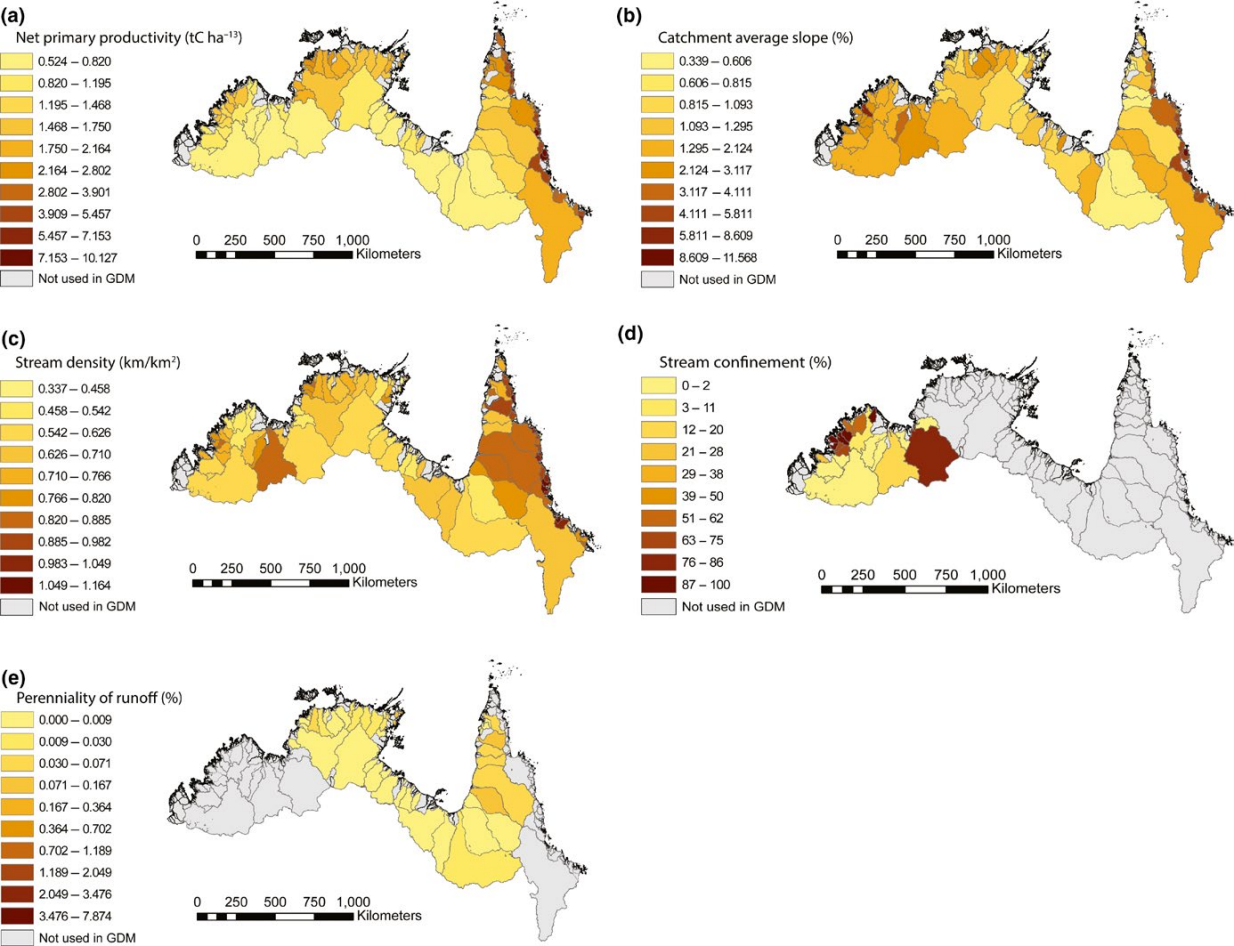
Visual representation of the environmental predictors selected by the generalised dissimilarity models (GDMs) for the Australian Monsoonal Tropics biome, and the Kimberley, Northern and Eastern provinces: (a) Net primary productivity (b) Catchment average slope (c) Stream density (d) Stream confinement (e) Perenniality of runoff. Environmental data is only shown for catchments used in GDMs (i.e., catchments with fish community composition data). Stream confinement and Perenniality of runoff were only significant predictors at the level of biogeographic province and as such, data is presented for the relevant province only

The model fit and nonlinear functions fitted to the predictor variables selected by the GDMs are shown in Figure 4. The patterns of change in the environmental variables selected by the models are visually presented in Figure 5. The plot of predicted ecological distance against observed compositional dissimilarity for the entire study region reflects the relatively high proportion of variance explained (64.5%) by the predictors. For the environmental predictor variables, the slope of the curve at any given point along the *x*‐axis of each of the graphs indicates the relative rate of compositional turnover detected by the analysis at that position along the environmental gradient in question. The total height reached by each function indicates the total amount of compositional turnover predicted to occur across the entire geographic range of the predictor, with all other predictors being constant. The most important predictors across the entire study region (AMT) were current geographical distance (number of catchment boundaries a fish must cross at current sea‐level heights), paleo‐geographical distance (number of paleo‐catchment boundaries a fish must cross at the maximum low sea level), catchment slope, Net Primary Productivity (NPP), and stream density. Geographical distance, NPP, catchment average slope and stream density displayed a relatively linear influence on species turnover while paleo‐geographical distance had the strongest affect on turnover at shorter distances (<35 catchments). Broadly speaking, catchment average slope was highest in Kimberley and Eastern, and was more variable across the Northern catchments. Catchments with the highest NPP were clustered around Northern Plateau and NW Plateau in Kimberley; Daly, Top End and Northern Gulf in Northern; and throughout Eastern. Catchments with the highest stream density were found throughout Eastern except for the northern and southern extremities, and higher stream densities were also clustered around Western Plateau, NW Plateau and Eastern Lowlands in Kimberley; and Daly, Top End and Northern Gulf in Northern.

The GDM for Kimberley Province performed best, explaining 65.1% of the variance in the data. The most important predictors were current geographical distance, paleo‐geographical distance, and catchment confinement. Current geographic distance affected species turnover at larger distances (>15 catchments), while paleo‐geographical distance had the greatest influence on species turnover at shorter distances (<9 catchments). Catchment confinement only affected turnover after a threshold of 23% (stream segments that flow through open plains rather than confined valleys) was reached. The most highly confined catchments were clustered around Western Plateau, NW Plateau and Northern Plateau, while the Victoria River was also highly confined.

The Northern Province GDM was the poorest performing, explaining 46.1% of the variance in the data. Geographic distance and perenniality of runoff were significant predictors of species turnover. Species turnover increased in a linear manner with current geographic distance and runoff perenniality. The northernmost catchments generally had higher runoff perenniality than those to the south. Catchments with the highest runoff perenniality were in Northern Gulf and the northeastern section of Southern Gulf.

The GDM for Eastern Province performed well, explaining 60.9% of the variance in the data. Current geographical distance and catchment average slope were the most important predictors of species turnover. Geographic distance increased linearly with species turnover, while catchment average slope had a positive curvilinear relationship with species turnover after a threshold of 4.3°. Catchment average slope was high across most Eastern catchments, but was notably lower around Cape York Peninsula and the Burdekin River in the Burdekin subprovince.

### 3.4 Inventory completeness index

Analysis of inventory completeness across the AMT (*C* = 0.90; 0.87–0.93) and its provinces (Northern: *C* = 0.93; 0.91–0.94, Eastern: *C* = 0.98; 0.96–1.00, and Kimberley: *C* = 0.85; 0.77–0.94) suggests the region has been well sampled, although the estimates indicated that Kimberley had the highest proportion of undetected species by far and the broadest confidence intervals (Table 3). The analysis of subprovinces again indicated quite high inventory completeness in all cases, although it was estimated that Southern Lowlands (*C* = 0.87; 0.80–0.94), NW Plateau (*C* = 0.86; 0.78–0.95), Northern Plateau (*C* = 0.79; 0.69–0.94), Eastern Lowlands (*C* = 0.88; 0.80–0.98), and Southern Gulf (*C* = 0.77; 0.69–0.88) subprovinces each had the largest proportion of undetected species, and they each had particularly wide confidence intervals (Table 3).

## 4 DISCUSSION

### 4.1 Freshwater fish biogeographic provinces of the AMT

The three freshwater fish provinces and 14 subprovinces proposed here significantly improve upon our previous understanding of the biogeography of the AMT. While our bioregionalisation scheme is in broad agreement with previous work by Unmack (2001, 2013), several key differences are apparent. At the broadest level, there are changes to the two former province boundaries within the AMT (Unmack, 2001). The main clusters of the WPGMA tree highlight a distinct split in species composition to the western side of Cape York Peninsula, below the Jardine River, and that at the tip of the peninsula and along the eastern side, representing the boundary of the newly expanded Eastern Province within the AMT. Surprisingly, we found the originally proposed Eastern‐Northern Province break north of the Burdekin River (Unmack, 2001, 2013) displayed species turnover typical of subprovince divides in the AMT, indicating the east coast drainages may form one continuous province that spans the east coast of Australia from the tip of Cape York Peninsula to Wilsons Promontory in Victoria (see Unmack (2001) for map of the southern boundaries of Eastern Province). The second change was a shift in the eastern boundary of Kimberley Province which now incorporates all drainages east of and including the Victoria River. The following are the generalised patterns within each province:

#### 4.1.1 Kimberley Province

The Kimberley Province has experienced, by far, the largest increase in knowledge of freshwater fish biodiversity (29 newly recognised species) and distributions since the last bioregionalisation (Unmack, 2001). As a result, the most significant changes in bioregionalisation were observed here. Most importantly, we extend the Kimberley Province from the Durack River east to the Fitzmaurice River, thus including an additional eight major river catchments and numerous coastal creeks. This expansion is further supported by phylogenetic analyzes of a number of widespread species that indicate that the lowland catchments between the Kimberley Plateau and the Fitzmaurice River are more strongly related to regions to the west than those to the east (Shelley, Swearer et al., 2018; Unmack, 2013; Unmack & Dowling, 2010).

The presence of such a strong biogeographic barrier for freshwater fish around the Joseph Bonaparte Gulf, seems counterintuitive as low sea‐level drainage patterns suggest that catchments between the King Edward River (Kimberley) and Howard River (Northern) were connected by a brackish lake during lowered sea levels (Yokoyama, Purcell, Lambeck, & Johnston, 2001). Furthermore, the presence of continuous and disjunct populations of multiple species that exist around the gulf (*Craterocephalus lentiginosus, Melanotaenia exquisita II, M. nigrans and Syncomistes bonapartensis*) suggest that there were dispersal pathways available for at least some freshwater fish species (Allen et al., 2002; Shelley, Swearer et al., 2018).

As the Wingate Ranges, a significant elevation divide, mark the Kimberley–Northern province boundary, it seems likely that ranges present a physical barrier to freshwater fish migration and are at least partially responsible for the divide. These ranges may have been passable during low sea levels via the coalescence of catchments or via Lake Bonaparte (depending on sea‐level heights), although: (a) habitat differences between the sandstone plateau highlands (Daly River side of the Wingate Ranges) and the alluvial lowland plains (Victoria River Basin side); (b) the disproportionate impact of aridity on the lowland southern catchments during glacial maxima (Pepper & Keogh, 2014); and (c) the brackish nature of Lake Bonaparte (Yokoyama et al., 2001) may have excluded many species from dispersing across the region at various points in time, thus reinforcing the barrier. Thus, it appears that while past freshwater connections across the Bonaparte Gulf have influenced species distributions, competing environmental and climatological influences may have led to the more complex biogeographic patterns observed today.

We also recognise six distinct subprovinces rather than the two proposed in Unmack (2001, 2013). These highly distinct and spatially confined subprovinces are defined by a high degree of narrow‐range endemism and fragmented populations of otherwise continuously distributed and widespread fishes observed across the Kimberley; 27 (66%) of the endemic species are restricted to one or two river systems (see Morgan et al., 2011 and Shelley, Morgan et al., 2018 for distributions). Of note, seven of the nine AMT catchments that were identified as focal points of endemism occurred around the Kimberley Plateau, with NW Plateau subprovince exhibiting the highest endemism in the AMT. It has been hypothesised that the topographical complexity of the Kimberley Plateau (see Figure 1) has acted to isolate freshwater fish populations across the landscape, contributing strongly to this phenomenon (Unmack, 2013).

On the other hand, Kimberley Province and its subprovinces are the least species rich in the AMT (78 species). This is largely due to a low proportion of widespread species (16 species, 21%) that have migrated from other regions. These findings are in line with more broad‐scale analyses of diversity in the AMT, which indicates the biogeographic boundaries that define Kimberley Province are particularly strong (Kennard, 2010; Unmack, 2001, 2013).

#### 4.1.2 Northern Province

Four broad subprovinces were identified across Northern Province, as compared to the nine identified by Unmack (2001, 2013). The main differences were the amalgamation of five smaller regions around the Gulf of Carpentaria into two larger subprovinces, Northern Gulf and Southern Gulf. The Northern Gulf is unique in that it is split across both sides of the Gulf of Carpentaria. This merging of the two regions is largely driven by the presence of species with fragmented populations on either side of the Gulf (*Pseudomugil gertrudae*,* Denariusa bandata*,* Melanotaenia trifasciata* III) and the mutual absence of species endemic to the Southern Gulf (*Parambassis gulliveri*,* Porochilus argenteus*,* Pingalla gilberti*). Recent phylogenetic work has determined the fragmented populations that occur on both sides of the Gulf have been connected within the last two to three glacial cycles, based on shared or similar haplotypes (Cook, Unmack, Huey, & Hughes, 2014; Huey et al., 2014; Unmack & Dowling, 2010). This relationship is most easily explained by drainage connections during low sea levels (Figure 1; Unmack, 2013). In the present Gulf of Carpentaria, there is a large depression that would have formed a lake containing fresh to brackish water during lowered sea levels. This feature, known as Lake Carpentaria, provided the potential for widespread connectivity across catchments draining into the Gulf, and those connections may have been present as recently as ~10,000 years ago (Reeves et al., 2008). However, this appears to contradict the division between the Northern and Southern Gulf subprovinces. A likely explanation for this is that the free dispersal of freshwater species via Lake Carpentaria was counteracted by increased aridity during glacial phases, particularly in the south of the AMT, which would have greatly reduced river perenniality and discharge into Lake Carpentaria from the Southern Gulf catchments (Playà, Cendón, Travé, Chivas, & García, 2007; Unmack, 2001). Geological evidence suggests that during the last glacial maximum, only the northernmost rivers of the Gulf (e.g., north of the Archer–Coen river on the eastern side) contributed water to Lake Carpentaria. Rivers in the south likely received less rainfall and would have had to flow further before entering the lake, and thus may have largely evaporated before reaching the lake (Playà et al., 2007), except perhaps during exceptional flood events.

Northern Province is the second most species rich (90 species), and equally most endemic (with Eastern) within the confines of the AMT (CWE = 0.68), although their endemism is still comparable with Kimberley Province (CWE = 0.64). Of note, the Top End has the second highest endemism of any subprovince in the AMT (CWE = 0.38), although no individual catchment was highlighted as being a focal point of endemism. This pattern reflects the wider distributions of endemic species that in turn lowers catchment‐scale CWE scores. The wider distribution of species (including endemics) across Northern Province is most readily explained by low sea‐level drainage patterns, which indicate that there has historically been broad hydrological connectivity between catchments draining into Lake Bonaparte and Lake Carpentaria, respectively. In Top End subprovince, low sea‐level drainage patterns indicate that catchments between the Adelaide and East Alligator rivers and between the Goomadeer and Goyder rivers were also hydrologically connected when the sea level was lowest (see Figure 1). Furthermore, drainage divides across the region are typically at low elevation near the current coastline (see Figure 1) and the catchments exhibit expansive lowland floodplains that may have provided opportunity for fish to move between basins via floodplain inundation during even slightly lowered sea levels.

Furthermore, Northern Province contains seven of the 11 most species rich catchments. While these catchments occur across each of the subprovinces, they form two broad clusters around the western Top End and Daly subprovinces and the tip of Cape York Peninsula. These findings reinforce general patterns highlighted in Kennard (2010) and Unmack (2001, 2013). Over half (48) of Northern Provinces’ species are shared with the island of New Guinea, which helps to explain the high richness and endemism values.

#### 4.1.3 Eastern Province

Eastern Province covers the tip of Cape York Peninsula and a long, narrow area that is wedged between the Great Dividing Range and the east coast of Australia. The four Eastern subprovinces were unchanged from those determined by Unmack (2001, 2013). The provincial divide (between Eastern and Northern) that our analysis highlights between the eastern and western sides of Cape York Peninsula, follows the Great Dividing Range to near its northern extent on the Australian mainland. At this point, the elevational divide is particularly low (see Figure 1), the opportunity for dispersal between the eastern and western sides would be much greater, and unsurprisingly the species composition is similar.

Eastern Province (within the AMT) has the most species (97 species), while endemism is equal with Northern Province (CWE = 0.68). The species richness in the province also benefits from the presence of 50 (52%) species that are shared with New Guinea and elsewhere in the Indo‐Pacific region. Wet Tropics is the most species rich (66 species) and endemic subprovince (CWE = 0.39) within Eastern province and across the AMT, with the Daintree and Mulgrave rivers being focal points of both richness and endemism. The Olive River in Cape York Peninsula is also a focal point of richness. Wet Tropics subprovince contains 13 Australian endemic species and a further 10 recently discovered amphidromous gobioid species that are locally endemic (i.e., within Australia) but also occur across the Indo‐Pacific (Thuesen et al., 2011). The defining features of Wet Tropics are the exceptionally high rainfall that leads to high reliability of flow, and steep catchment gradients that, in combination with the high and consistent flow, produce an array of perennial lotic habitats that are unique within the ATM and Australia in general. Some rivers draining Cape York Peninsula, such as the Olive River, provide similar conditions. These factors have been hypothesised to be reasons for the high species richness in the Wet Tropics (Pusey, Arthington, & Read, 1995; Pusey and Kennard, 1996; Unmack, 2001), and the presence of many amphidromous gobioids that have strong habitat associations with fast flowing, perennial habitat (Keith, 2003) provides strong support. We believe that the same reasoning can be applied to the Olive River.

### 4.2 Linking freshwater fish species turnover to the environment

Here, we provide the first attempt to link turnover in catchment‐scale freshwater fish species composition with measures of current and paleo‐geographical distance, and broad‐scale environmental variables. Our GDM analysis determined that variation in freshwater fish species composition across the AMT was best explained by increases in current and paleo‐geographical distance between catchments, catchment average slope, terrestrial NPP, and stream density.

The identification of paleo‐geographical distance as a significant factor influencing species turnover supports strong genetic evidence for the influence of low sea‐level drainage patterns on species and population connectivity in the region (Baker et al., 2008; Cook et al., 2012; De Bruyn et al., 2004; Huey et al., 2014). The widespread and/or disjunct species distributions of species around the paleo‐lakes discussed above are the clearest examples of this, although smaller‐scale cases of increased connectivity between catchments during lowered sea levels are also present across the entire region.

Given the AMT is a largely flat and geologically stable landscape (Johnson, 2004) it is unsurprising that elevation changes, which influence catchment slope, would also have a strong influence on species distributions. The Kimberley Plateau (maximum elevation 854 m), Eastern Highlands (1,355 m), and to a lesser extent the Arnhem Plateau (450 m) and mountain ranges that form the AMT's southern boundary, are the region's prominent high elevation features (Figure 1). They provide high relief, steeply sloped aquatic habitat that typically takes the form of fast flowing, shallow, rocky bottom streams that lie in contrast to the large, sluggish lowland reaches (Gordon, McMahon, & Finlayson, 2004). When these high elevation features are close to the coastline the catchments that drain them are more steeply sloped, smaller, and have little or no lowland habitat. These areas are particularly concentrated around the northern Kimberley Plateau and between Cape York Peninsula and Burdekin subprovinces in Eastern Province. Such features would present significant dispersal barriers to a number of species (Albert, Petry, & Reis, 2011).

The measures of terrestrial NPP reflect biological (i.e., plant) responses to the light, thermal and moisture regimes (Kennard, 2010). Various factors influence such productivity although rainfall and temperature are the most important (Eamus, 2003). A simple interpretation is that hot and wet areas (e.g., tropical rain forests and coastal wetlands) tend to have high NPP. Stream density indicates the amount and nature of aquatic habitat available and is also heavily influenced by rainfall in addition to the physical characteristics of the catchment such as catchment slope, soil permeability and the underlying rock type (Pidwirny, 2006). Regions with high rainfall, high relief catchments and impermeable ground or exposed bedrock tend to have higher runoff and therefore higher stream density. Stream density of a catchment influences its hydraulic response to rainfall events, with catchments with high stream density exhibiting rapid rises and falls in flows (Carlston, 1963).

As NPP and stream density reflect multiple, intertwining biological and physical processes, they may present more ecologically relevant measures of climate effects on freshwater ecosystems in this context than simple rainfall or air temperature data. Broadly speaking, the catchments with the highest NPP and stream density were mostly to the north of the AMT and along the east coast (e.g., Western Plateau and NW Plateau in Kimberley; Daly, Top End and Northern Gulf in Northern and throughout Eastern) where the terrain is rugged and sloped, rainfall is higher and wet‐season temperatures are lower than the southern regions of Kimberley and Northern. For instance, the northern and eastern coastlines receive annual median rainfalls >1,500 mm and experience mean wet‐season temperatures <35°C, while more inland regions receive annual median rainfall as low as 300 mm and experience mean wet‐season temperatures >35°C (Australian Government Bureau of Meteorology; www.bom.gov.au/climate/change/acorn-sat/). As proposed by Unmack (2001, 2013), these gradients present likely biological filters confining mesic adapted species to the north and east coast, while only arid adapted species may occur in the south. This was exemplified in a study of biogeographic determinants of life‐history indices in Australian freshwater fishes (Sternberg & Kennard, 2014), which found that NPP and associated variables such as temperature and runoff were important for explaining variation in the frequency and distribution of freshwater fish life‐history strategies in Australian river basins. In a similar study investigating environmental drivers of specific life‐history traits, Sternberg and Kennard (2013) identified strong associations across AMT river basins between fish exhibiting traits associated with the “periodic” endpoint strategy (Winemiller & Rose, 1992; e.g., large bodied, late maturing, broadcast spawners that produce large numbers of small eggs) with river basins dominated by high mean annual temperature and low perenniality (e.g., the most southern subprovinces). On the other hand, fish exhibiting traits associated with the “equilibrium” strategy (i.e., larger eggs, lower fecundity and intermediate age and length at maturity) were more frequent in environments typified by a high NPP, high gradient, high stream density, and low variation in mean annual temperature (e.g., the most northern subprovinces and much of the Eastern Province). This may also help explain why no subprovince encompasses both northern and southern catchments. While the physiological tolerances of AMT fishes are poorly known, this is a likely explanation for the restriction of many Eleotridae, Gobiidae and Melanotaeniidae species to Top End, Northern Gulf and north of Burdekin along the east coast.

Within Kimberley Province, current and paleo‐geographical distance, and catchment confinement (Figure 5e) best explained species turnover. This aligns with our expectations as the catchments draining the Kimberley Plateau are highly constrained by the region's rugged topography limiting fish migration between catchments under current sea‐level heights and driving high species turnover over short geographic distances (Huey et al., 2014; Phillips, Storey, & Johnson, 2009; Shelley, Swearer et al., 2018). However, lowered sea levels exposed a wide continental shelf and some of the regions currently isolated rivers coalesced before reaching the ocean providing freshwater fish species an opportunity to expand their distributions to varying degrees. Therefore, it is also expected that the closer proximity or connectivity of these highly isolated catchments under low sea levels would have a particularly strong influence of community composition similarity between impacted catchments. The clearest example of broadened connectivity during low sea levels is observed in the catchments that would have drained into Lake Bonaparte (Yokoyama et al., 2001). However, smaller‐scale low sea‐level connections, such as between the Prince Regent and Roe rivers (NW Plateau), also provide an explanation for the similarity of species composition between some plateau catchments.

Across Northern Province, current geographical distance and runoff perenniality best explained species turnover, based on our model. Runoff perenniality is largely driven by the prevailing rainfall, temperature and geology (Seaman et al., 2016). As such, the declining north to south gradient in perenniality broadly reflects the patterns observed in NPP and natural forest cover. This gradient provides a reasonable explanation for the divide between Northern Gulf and Southern Gulf and Top End and Daly as any exacerbation of the length and frequency of stream drying during the dry‐season would act to filter out species adapted to the more benign conditions experienced in streams with greater perenniality, as discussed above. Another explanation for the divides between Daly, Top End, and Northern Gulf subprovinces are differences in the orientation of the drainages that flow west, north, and east, respectively, off the Arnhem Plateau (Figure 1). Low sea‐level drainage patterns suggest that these subprovinces would have historically flowed into Lake Bonaparte (Daly), off the north coast (Top End), and into Lake Carpentaria (Northern Gulf) and as such the degree of isolation between their respective catchments (i.e., between river mouths) appears to have remained high throughout the glacial cycles and may have reinforced faunal differences.

Turnover in species composition across Eastern Province was most heavily influenced by current geographical distance and catchment average slope (Figure 5b). This is unsurprising given the short catchments drain directly from the Great Dividing Range into the ocean. Even small variations in the height and direction of the mountain range strongly influence catchment slope, causing corresponding changes in habitat and catchment connectivity as discussed above. Furthermore, the continental shelf off the east coast is narrow and straight, so it was expected that that lowered sea levels would have less of an influence on species distributions in Eastern (Unmack, 2013).

### 4.3 Identification of sampling gaps

Having assembled a comprehensive checklist of freshwater fishes across the AMT, we investigated the completeness of the inventory to identify under‐sampled regions. We also mapped which rivers lacked published species‐composition data. As a whole, the AMT and its major provinces were broadly considered to be well sampled, although Kimberley Province, and Southern Lowlands, NW Plateau, Northern Plateau, Eastern Lowlands and Southern Gulf subprovinces were estimated to have the highest number of undetected species. The results largely reflect the difficulty in accessing these more remote areas. The lower sampling effort in Kimberley Province is further highlighted by the presence of major catchments, such as the Berkley and Forrest rivers that lack fish composition data. These are particularly critical gaps as they lie on the border between Northern Plateau and Eastern Lowland subprovinces, so their true boundaries cannot be determined. Another significant data gap exists at the Nesbit River between Lakefield and Cape York Peninsula subprovinces that needs to be filled to determine the inter‐province boundary. Finally, a number of catchments between the Habgood and Roper rivers lack species‐composition data and further sampling is needed to clarify patterns of freshwater fish biodiversity in the area.

### 4.4 How do patterns in freshwater fish biodiversity compare with other taxonomic groups?

It has become clear that there are distinct patterns of endemism, species distribution and range sizes across plant and animal taxonomic groups that form remarkably coherent biogeographic patterns in the AMT. It appears that these patterns reflect a shared biological response to the region's unique physical environment and geological history.

The AMT encompasses four broad areas of endemism referred to as the Kimberley Plateau, Arnhem Land, Cape York Peninsula, and the Atherton Tablelands (similar to the Wet Tropics subprovince; Cracraft, 1991; Crisp, Linder, & Weston, 1995). Each of these areas is centered on the three major upland regions: the Kimberley Plateau, Arnhem Plateau and the Great Dividing Range (reviewed in Bowman et al., 2010). In a grid‐based analysis of species distributions, Ebach, Laffan, and Cassis (2017) demonstrated that this pattern is common across terrestrial plant, vertebrate, invertebrate and fungi groups. There is a broad consensus that these upland regions provided mesic refuge during periods of extreme aridity, driven by late Pliocene and Pleistocene glacial cycles (Byrne et al., 2008; Fujioka & Chappell, 2010). Species would have contracted to these refuges and while isolated in these geographically complex landscapes, some appear to have diversified at particularly fine geographic scales. Phylogenetic research has revealed hotspots of cryptic, narrow‐range species associated with the Kimberley Plateau, Arnhem Plateau and Wet Tropics in a range of taxonomic groups including mammals (Bowman et al., 2010; Potter, Eldridge, Taggart, & Cooper, 2012), lizards (Fujita, McGuire, Donnellan, & Moritz, 2010; Oliver, Adams, & Doughty, 2010; Melville, Ritchie, Chapple, Glor, & Schulte, 2011; Smith, Harmon, Shoo, & Melville, 2011), frogs (Slatyer, Rosauer, & Lemckert, 2007), and invertebrates (Yeates, Bouchard, & Monteith, 2002).

Areas of high endemism in freshwater fish overlap with those centered on the Wet Tropics, and Kimberley and Arnhem plateaus, with the subprovinces with the highest CWE scores being Wet Tropics (Atherton Tablelands), Top End (Arnhem Plateau), and NW Plateau (Kimberley Plateau), respectively. However, narrow‐range endemism was most strongly focussed around the Kimberley Plateau and Wet Tropics, where all focal points of endemism were identified. These patterns of narrow‐range endemism have been highlighted by recent surveys, and phylogenetic and taxonomic studies (Hammer et al., 2018; Shelley, Morgan et al., 2018; Shelley, Swearer et al., 2018; Thuesen et al., 2011). An excellent example of the influence these rugged upland regions may have on patterns on patterns of diversification can be found within Australia's third most widespread freshwater fish, *Amniataba percoides*. Recently, *A. percoides* has been proposed to comprise at least four candidate cryptic species within the Kimberley, but only one across the rest of its range that spans approximately two‐thirds of Australia (Shelley, Swearer et al., 2018). In contrast, endemism in freshwater fish is relatively low around Cape York Peninsula. Broad connectivity around Lake Carpentaria during lowered sea levels (including with New Guinea) and greater opportunity for connectivity with the east coast drainages likely provided greater opportunity for wider dispersal and, as a result, there is less geographically constrained endemism.

A number of biogeographic barriers have been identified across the AMT areas based on a combination of climatic and topological factors (reviewed in Bowman et al., 2010; Catullo, Lanfear, Doughty, & Keogh, 2014; Eldridge, Potter, & Cooper, 2011). However, the most notable barriers occur in the arid, lowland areas referred to here as the Kimberley–Arnhem Land Barrier (sensu Eldridge et al., 2011) and Carpentarian Gap (sensu MacDonald, 1969). The Kimberley–Arnhem Land Barrier covers the broad lowland region between the Kimberley and Arnhem plateaus within which multiple taxa specific barriers have been identified (Eldridge et al., 2011). On the other hand, the Carpentarian Gap is a more discrete, well‐defined region between the Flinders and Norman rivers in the southern Gulf of Carpentaria (Catullo et al., 2014). These barriers present significant population‐ and/or species‐level divides for many taxonomic groups (e.g., plants: Crisp et al., 1995; González‐Orozco et al., 2014; terrestrial vertebrates: Catullo et al., 2014; Cracraft, 1991; Potter et al., 2012; invertebrates: Matthews & Bouchard, 2008). Cladistics analyses of terrestrial vertebrate (Cracraft, 1991) and plant groups (Crisp et al., 1995) indicate that these barriers divide the AMT into three broad phytogeographical and zoogeographical subregions (analogous to our Provinces) that have been described around the Kimberley Plateau, Arnhem Land and Cape York Peninsula areas of endemism. The Atherton Tablelands (similar to our Wet Tropics subprovince) is also classified as a subregion on the east coast, with the boundary being defined by lower elevation landscapes to the north and by drier, open woodland to the south (known as the Burdekin Gap), which also form the southern boundary of the AMT.

These analyses (Cracraft, 1991; Crisp et al., 1995) have been adopted into the provisional Australian Bioregionalisation Atlas presented in Ebach et al. (2013) that presents a basis for comparison with the broad biogeographic provinces determined by our analysis. In the case of freshwater fish, the Kimberley Province overlaps exactly with the phytogeographical and zoogeographical subregions (i.e., Kimberley Plateau) defined in Ebach et al. (2013), with the Wingate Ranges forming the provinces eastern boundary. However, the Northern Province is unique in that it encompasses the Arnhem Land subregion as well as part of the Cape York Peninsula subregion; the western side of the Great Dividing Range (as far north as the Wenlock River). The simplest explanation for this is that freshwater fishes could circumvent the arid Carpentarian Gap by crossing Lake Carpentaria (elaborated on in the Northern Province section of the Discussion), while terrestrial taxonomic groups would still have to disperse around the lake, across the arid region. Conversely, the Great Dividing Range could be more easily crossed by terrestrial taxa than freshwater fish that are largely bound to river catchments. Therefore, the Northern and Eastern freshwater fish biogeographic provinces are divided on either side of the Great Dividing Range, while the phytogeographical and zoogeographical subregions span both sides. Finally, the Atherton Tablelands subregion overlaps closely with the Wet Tropics subprovince highlighted in our analysis spanning the region from the Bloomfield River to just north of the Burdekin River catchment. This is unsurprising given the exceptionally high rainfall and strong elevation gradient that shapes the distinct freshwater habitat in the region and in turn supports its unique fish fauna, also supports the region's unique enclosed rainforests and associated endemic terrestrial fauna (Williams et al., 2010).

Overall, freshwater fish biogeographic patterns support many of the major patterns present in the AMT, while exhibiting some that are likely unique to obligate freshwater organisms. The impact of geological barriers (e.g., the Great Dividing Range) for instance, appears to have a far greater impact of freshwater fish distribution than on terrestrial plant and animal groups. Conversely, the large paleo‐lakes likely facilitated dispersal of freshwater fishes, but not terrestrial taxa. Freshwater fishes thus provide important, alternative insight into understanding the complex biogeographic history of the region.

## 5 CONCLUSIONS

Our provinces present a number of significant changes to previous broad‐scale studies of freshwater fish species composition in the AMT that were based on a much smaller dataset (Unmack, 2001, 2013). Key differences in our study include changes to the Kimberley−Northern and Northern−Eastern province boundaries and a major refinement of subprovinces of Kimberley Province in light of substantial increases in sampling and taxonomic knowledge for the area (Morgan et al., 2011; Shelley, Morgan et al., 2018). We found that the influence of high elevation geological features, such as the Kimberley and Arnhem plateaus and Eastern Highlands, on aquatic habitat as well as stream density and light, thermal and moisture regimes are the most important environmental factors influencing species turnover. Historic low sea‐level drainage patterns were also influential, highlighting the importance of history in explaining contemporary patterns of biodiversity in the AMT.

Our analysis of richness and endemism using the larger data set mostly reinforced previously described patterns (Kennard, 2010; Unmack, 2001, 2013). The Daintree and Mulgrave catchments in the Wet Tropics are unique in that they are focal points of both narrow‐range endemism and species richness and are thus of great conservation importance. The catchments surrounding the Kimberley Plateau also represent focal points of outstanding narrow‐range endemism.

The patterns of freshwater fish diversity identified here provide a foundation for future biogeographic studies. Continued sampling, especially in the regions that we identified as poorly sampled or data deficient, is necessary to refine our proposed provinces. A thorough understanding of the basis for these patterns as well as the environmental/habitat preferences of the freshwater fish fauna of the AMT are vital avenues of further research to help inform conservation planning decisions.

## CONFLICT OF INTEREST

None declared.

## AUTHOR CONTRIBUTIONS

J.J.S. and S.E.S. conceived the ideas; M.C.L., J.J.S., M.C.L and P.J.U. collected the data; J.J.S. analysed the data under the supervision of S.W.L.; J.J.S. led the writing, and all authors contributed through editing.

## Data Availability

Data relating to fish distributions in the study catchments can be found in the Dryad database (https://doi.org/10.5061/dryad.330tj7f).
