# Supplementary material for: A revision of the bioregionalisation of freshwater fish communities in the Australian Monsoonal Tropics
Source: Ecol Evol. 2019 Mar 29;9(8):4568–88. doi: 10.1002/ece3.5059 (PMC6476826; doi:10.1002/ece3.5059)
Supplement: Supplementary file 1 [file ECE3-9-4568-s001.docx]

*Ecology and Evolution*

**SUPPORTING INFORMATION**

A revision of the bioregionalisation of freshwater fish communities in the Australian Monsoonal Tropics

**James J. Shelley^1*^, Tim Dempster^1^, Matthew C. Le Feuvre^1^, Peter J. Unmack^2^, Shawn W. Laffan^3^, Stephen E. Swearer^1^**

Appendix 1 Fish distribution data sources

Appendix 2 Candidate species used in this study, the morphological and genetic evidence from which their candidate species status was determined, and the references for that evidence

Appendix 3 List of described and candidate freshwater bony fishes and the provinces and subprovinces in which they are found

Appendix 1

**Kimberley Province** (the key sources used were Morgan et al. (2011) and Shelley et al. (2018a) and with additional survey data sources listed after)

Morgan D.L., Allen G.R., Pusey B.J., & Burrows D.W. (2011) A review of the freshwater fishes of the Kimberley region of Western Australia. *Zootaxa*, **2816**, 1–64.

Hammer M., Museum and Art Gallery of the Northern Territory, Unpublished survey data from the Victoria and Fitzmaurice rivers.

Moore G. I. & Hammer, M. P. (2015). Freshwater fishes of three tributaries of the Pentecost River, Kimberley, Western Australia. *Records of the Western Australian Museum*, **30**, 60–71.

Pusey B.J., Burrows D.W., Kennard M.J., Perna C.N., Unmack P.J., Allsop Q., & Hammer M.P. (2017) Freshwater fishes of northern Australia. *Zootaxa*, **4253(1)**, 1–104.

Shelley J.J., Delaval D., & Le Feuvre M.C. (2017) A revision of the grunter genus Syncomistes (Teleostei, Terapontidae, Syncomistes) with descriptions of seven new species from the Kimberley region, northwestern Australia. *Zootaxa*, **4367(1)**, 1–103.

Shelley J.J., Swearer S.E., Adams M., Dempster T., Le Feuvre M.C., Hammer M.P., & Unmack P.J. (2018b) Cryptic biodiversity in the freshwater fishes of the Kimberley endemism hotspot, northwestern Australia. *Molecular Phylogenetics and Evolution*, **127**, 843–858.

**Northern Province** (the key source used was Pusey et al., 2017 with additional survey data sources listed after)

Pusey B.J., Burrows D.W., Kennard M.J., Perna C.N., Unmack P.J., Allsop Q., & Hammer M.P. (2017) Freshwater fishes of northern Australia. *Zootaxa*, **4253(1)**, 1–104.

Smith R., Hydrobiology Pty Ltd., Unpublished survey data from Norman Creek and the Watson and Embley rivers.

**Eastern Province** (the key source used was Pusey et al., 2004 with additional survey data sources listed after)

Pusey B.J., Kennard M.J. & Arthington A.H. (2004) Freshwater Fishes of North-eastern Australia. CSIRO Publishing, Melbourne.

Atlas of Living Australia (2013).

Ebner B.C., Kroll B., Godfrey P., Thuesen P.A., Vallance T., Pusey B., Allen G.R., Rayner T.S., & Perna C.N. (2011) Is the elusive Gymnothorax polyuranodon really a freshwater moray? *Journal of Fish Biology*, **79**, 70–79.

Ebner B.C., James Cook University, Unpublished survey data from the Bloomfield River.

Johnson J.W. & Randall J.E. (2008) *Synclidopus hogani*, a new species of soleid fish from northeastern Queensland, Australia. *Memoirs of the Queensland Museum*, **52**, 245–254.

Hammer, M. P., Allen, G. R., Martin, K. C., Adams, M. A. R. K., Ebner, B. C., Raadik, T. A., & Unmack, P. J. (2018). Revision of the Australian Wet Tropics endemic rainbowfish genus *Cairnsichthys* (Atheriniformes: Melanotaeniidae), with description of a new species. *Zootaxa*, **4413(2)**, 271–294.

Thuesen P.A., Ebner B.C., Larson H., Keith P., Silcock R.M., Prince J., & Russell D.J. (2011) Amphidromy links a newly documented fish community of continental Australian streams, to oceanic islands of the west Pacific. *PLoS ONE*, **6**, e26685.

Appendix 2

| **Species** | **References** | **Morphological and genetic evidence** |
| --- | --- | --- |
| *Neosilurus hyrtlii* I | Huey et al. (2014), P. Unmack et al. (unpublished genetic data), M. Adams (unpublished genetic data) | *Mitochondrial Markers*: ATP6, ATP8, and COIII genes (total 694 bp), 18S (1797 bp), five protein coding mitochondrial genes (ND4L, ND4, ND5, ND6, cytb, total 5159 bp). *Nuclear Markers*: RAG1 (1075 bp), cytb (1140 bp), 54 allozyme loci. |
| *Neosilurus hyrtlii* II | Huey et al. (2014), P. Unmack et al. (unpublished genetic data), M. Adams (unpublished genetic data) | *Mitochondrial Markers*: ATP6, ATP8, and COIII genes (total 694 bp), 18S (1797 bp), five protein coding mitochondrial genes (ND4L, ND4, ND5, ND6, cytb, total 5159 bp). *Nuclear Markers*: RAG1 (1075 bp), cytb (1140 bp), 54 allozyme loci. |
| *Neosilurus hyrtlii* III | Huey et al. (2014), P. Unmack et al. (unpublished genetic data), M. Adams (unpublished genetic data) | *Mitochondrial Markers*: ATP6, ATP8, and COIII genes (total 694 bp), 18S (1797 bp), five protein coding mitochondrial genes (ND4L, ND4, ND5, ND6, cytb, total 5159 bp). *Nuclear Markers*: RAG1 (1075 bp), cytb (1140 bp), 54 allozyme loci. |
| *Neosilurus pseudospinosus* I | Huey et al. (2014), P. Unmack et al. (unpublished genetic data), M. Adams (unpublished genetic data) | *Mitochondrial Markers*: ATP6, ATP8, and COIII genes (total 694 bp), 18S (1797 bp), five protein coding mitochondrial genes (ND4L, ND4, ND5, ND6, cytb, total 5159 bp). *Nuclear Markers*: RAG1 (1075 bp), cytb (1140 bp), 54 allozyme loci. |
| *Neosilurus pseudospinosus* II | Huey et al. (2014), P. Unmack et al. (unpublished genetic data), M. Adams (unpublished genetic data) | *Mitochondrial Markers*: ATP6, ATP8, and COIII genes (total 694 bp), 18S (1797 bp), five protein coding mitochondrial genes (ND4L, ND4, ND5, ND6, cytb, total 5159 bp). *Nuclear Markers*: RAG1 (1075 bp), cytb (1140 bp), 54 allozyme loci. |
| *Neosilurus* sp. (Flinders) | Hogan and Vallance (2005), P. Unmack et al. (unpublished genetic data) | Morphology appraisal. *Mitochondrial Markers*: five protein coding mitochondrial genes (ND4L, ND4, ND5, ND6, cytb, total 5159 bp), 18S (1797 bp). *Nuclear Markers*: RAG1 (1075 bp). |
| *Craterocephalus lentiginosus* I | P. Unmack (unpublished genetic data) | *Mitochondrial Marker*: cytb (1140 bp) |
| *Craterocephalus lentiginosus* II | P. Unmack (unpublished genetic data) | *Mitochondrial Marker*: cytb (1140 bp) |
| *Craterocephalus lentiginosus* III | P. Unmack (unpublished genetic data) | *Mitochondrial Marker*: cytb (1140 bp) |
| *Craterocephalus stercusmuscarum* I | Unmack and Dowling (2010) | *Mitochondrial Marker*: cytb (1140 bp) |
| *Craterocephalus stercusmuscarum* II | Unmack and Dowling (2010) | *Mitochondrial Marker*: cytb (1140 bp) |
| *Craterocephalus stercusmuscarum* III | Unmack and Dowling (2010) | *Mitochondrial Marker*: cytb (1140 bp) |
| *Craterocephalus stramineus* I | Unmack and Dowling (2010) | *Mitochondrial Marker*: cytb (1140 bp) |
| *Craterocephalus stramineus* II | Unmack and Dowling (2010) | *Mitochondrial Marker*: cytb (1140 bp) |
| *Craterocephalus stramineus* III | Unmack and Dowling (2010) | *Mitochondrial Marker*: cytb (1140 bp) |
| *Iriatherina werneri* I | Unmack et al. (2013) | *Mitochondrial Markers*: Seven protein coding mitochondrial genes (ND1, ND2, ATPase 8, ATPase 6, ND4L, ND4, Cytb, total 5696 bp). *Nuclear Markers*: S7 (total 1130 bp), SNPs (~10000 loci). |
| *Iriatherina werneri* II | Unmack et al. (2013) | *Mitochondrial Markers*: Seven protein coding mitochondrial genes (ND1, ND2, ATPase 8, ATPase 6, ND4L, ND4, Cytb, total 5696 bp). *Nuclear Markers*: S7 (total 1130 bp), SNPs (~10000 loci). |
| *Melanotaenia exquisita* I | P. Unmack & M. Hammer (unpublished genetic data) | *Mitochondrial Markers*: Seven protein coding mitochondrial genes (ND1, ND2, ATPase 8, ATPase 6, ND4L, ND4, Cytb, total 5696 bp). *Nuclear Markers*: S7 (total 1130 bp), SNPs (~10000 loci). |
| *Melanotaenia exquisita* II | P. Unmack & M. Hammer (unpublished genetic data) | *Mitochondrial Markers*: Seven protein coding mitochondrial genes (ND1, ND2, ATPase 8, ATPase 6, ND4L, ND4, Cytb, total 5696 bp). *Nuclear Markers*: S7 (total 1130 bp), SNPs (~10000 loci). |
| *Melanotaenia maccullochi* I | Unmack et al. (2013), Cook et al. (2014), P. Unmack et al. (unpublished genetic data) | *Mitochondrial Markers*: Seven protein coding mitochondrial genes (ND1, ND2, ATPase 8, ATPase 6, ND4L, ND4, Cytb, total 5696 bp). *Nuclear Markers*: S7 (total 1130 bp), SNPs (~10000 loci). |
| *Melanotaenia maccullochi* II | Unmack et al. (2013), Cook et al. (2014), P. Unmack et al. (unpublished genetic data) | *Mitochondrial Markers*: Seven protein coding mitochondrial genes (ND1, ND2, ATPase 8, ATPase 6, ND4L, ND4, Cytb, total 5696 bp). *Nuclear Markers*: S7 (total 1130 bp), SNPs (~10000 loci). |
| *Melanotaenia maccullochi* III | Unmack et al. (2013), Cook et al. (2014), P. Unmack et al. (unpublished genetic data) | *Mitochondrial Markers*: Seven protein coding mitochondrial genes (ND1, ND2, ATPase 8, ATPase 6, ND4L, ND4, Cytb, total 5696 bp). *Nuclear Markers*: S7 (total 1130 bp), SNPs (~10000 loci). |
| *Melanotaenia trifasciata* I | Unmack et al. (2013), P. Unmack et al. (unpublished genetic data) | *Mitochondrial Markers*: Seven protein coding mitochondrial genes (ND1, ND2, ATPase 8, ATPase 6, ND4L, ND4, Cytb, total 5696 bp). *Nuclear Markers*: S7 (total 1130 bp), SNPs (~10000 loci). |
| *Melanotaenia trifasciata* II | Unmack et al. (2013), P. Unmack et al. (unpublished genetic data) | *Mitochondrial Markers*: Seven protein coding mitochondrial genes (ND1, ND2, ATPase 8, ATPase 6, ND4L, ND4, Cytb, total 5696 bp). *Nuclear Markers*: S7 (total 1130 bp), SNPs (~10000 loci). |
| *Melanotaenia trifasciata* III | Unmack et al. (2013), P. Unmack et al. (unpublished genetic data) | *Mitochondrial Markers*: Seven protein coding mitochondrial genes (ND1, ND2, ATPase 8, ATPase 6, ND4L, ND4, Cytb, total 5696 bp). *Nuclear Markers*: S7 (total 1130 bp), SNPs (~10000 loci). |
| *Melanotaenia trifasciata* IV | Unmack et al. (2013), P. Unmack et al. (unpublished genetic data) | *Mitochondrial Markers*: Seven protein coding mitochondrial genes (ND1, ND2, ATPase 8, ATPase 6, ND4L, ND4, Cytb, total 5696 bp). *Nuclear Markers*: S7 (total 1130 bp), SNPs (~10000 loci). |
| *Pseudomugil signifer* I | McGlashan & Hughes (2002), Wong et al. (2004) | *Mitochondrial Marker*: ATP6 (633 bp). *Nuclear Markers*: 6 allozyme loci. |
| *Pseudomugil signifer* II | McGlashan & Hughes (2002), Wong et al. (2004) | *Mitochondrial Marker*: ATP6 (633 bp). *Nuclear Markers*: 6 allozyme loci. |
| *Ambassis* sp. 2 | P. Unmack & D. Morgan (unpublished genetic and morphological data). Also see Shelley et al. (2018a) for morphological appraisal. | Morphological appraisal, *Mitochondrial Marker*: cytb (1140 bp). *Nuclear Marker* S7 (921 bp). |
| *Amniataba* sp. 1 | Shelley et al. (2018a), J. Shelley & M. Le Feuvre (unpublished morphological data). Also see Shelley et al. (2018b) for morphological appraisal. | *Morphological analysis* (56 characters), *Mitochondrial Marker*: cytb (601 bp). *Nuclear Markers*: 54 allozyme loci, RAG1 (2541 bp). |
| *Amniataba percoides* I | Shelley et al. (2018a) | *Mitochondrial Marker*: cytb (601 bp). *Nuclear Markers*: 54 allozyme loci, RAG1 (2541 bp). |
| *Amniataba percoides* II | Shelley et al. (2018a) | *Mitochondrial Marker*: cytb (601 bp). *Nuclear Markers*: 54 allozyme loci, RAG1 (2541 bp). |
| *Amniataba percoides* III | Shelley et al. (2018a) | *Mitochondrial Marker*: cytb (601 bp). *Nuclear Markers*: 54 allozyme loci, RAG1 (2541 bp). |
| *Amniataba percoides* IV | Shelley et al. (2018a) | *Mitochondrial Marker*: cytb (601 bp). *Nuclear Markers*: 54 allozyme loci, RAG1 (2541 bp). |
| *Amniataba percoides* V | Shelley et al. (2018a) | *Mitochondrial Marker*: cytb (601 bp). *Nuclear Markers*: 54 allozyme loci, RAG1 (2541 bp). |
| *Hannia greenwayi* I | Shelley et al. (2018a), J. Shelley & M. Le Feuvre (unpublished morphological data). Also see Shelley et al. (2018b) for morphological appraisal. | *Morphological analysis* (56 characters), *Mitochondrial Marker*: cytb (601 bp). *Nuclear Markers*: 54 allozyme loci, RAG1 (2541 bp). |
| *Hannia greenwayi* II | Shelley et al. (2018a), J. Shelley & M. Le Feuvre (unpublished morphological data). Also see Shelley et al. (2018b) for morphological appraisal. | *Morphological analysis* (56 characters), *Mitochondrial Marker*: cytb (601 bp). *Nuclear Markers*: 54 allozyme loci, RAG1 (2541 bp). |
| *Glossamia aprion* I | Cook et al. (2017) | *Mitochondrial Marker*: ATP6 (615 bp). *Nuclear Markers*: 38 allozyme loci. |
| *Glossamia aprion* II | Cook et al. (2017) | *Mitochondrial Marker*: ATP6 (615 bp). *Nuclear Markers*: 38 allozyme loci. |
| *Glossamia aprion* III | Cook et al. (2017) | *Mitochondrial Marker*: ATP6 (615 bp). *Nuclear Markers*: 38 allozyme loci. |
| *Glossamia aprion* IV | Cook et al. (2017) | *Mitochondrial Marker*: ATP6 (615 bp). *Nuclear Markers*: 38 allozyme loci. |
| *Hypseleotris ejuncida* I | J. Shelley, P. Unmack & C. Thacker (unpublished genetic data), J. Shelley (unpublished morphological data). Also see Shelley et al. (2018b) for morphological appraisal. | *Morphological analysis* (46 characters), *Mitochondrial Marker*: cytb (1140 bp). *Nuclear Markers*: ~500 UCE loci. |
| *Hypseleotris ejuncida* II | J. Shelley, P. Unmack & C. Thacker (unpublished genetic data), J. Shelley (unpublished morphological data). Also see Shelley et al. (2018b) for morphological appraisal. | *Morphological analysis* (46 characters), *Mitochondrial Marker*: cytb (1140 bp). *Nuclear Markers*: ~500 UCE loci. |
| *Hypseleotris kimberleyensis* I | J. Shelley, P. Unmack & C. Thacker (unpublished genetic data), J. Shelley (unpublished morphological data). Also see Shelley et al. (2018b) for morphological appraisal. | *Morphological analysis* (46 characters), *Mitochondrial Marker*: cytb (1140 bp). *Nuclear Markers*: ~500 UCE loci. |
| *Hypseleotris kimberleyensis* II | J. Shelley, P. Unmack & C. Thacker (unpublished genetic data), J. Shelley (unpublished morphological data). Also see Shelley et al. (2018b) for morphological appraisal. | *Morphological analysis* (46 characters), *Mitochondrial Marker*: cytb (1140 bp). *Nuclear Markers*: ~500 UCE loci. |
| *Hypseleotris regalis* I | J. Shelley, P. Unmack & C. Thacker (unpublished genetic data), J. Shelley (unpublished morphological data). Also see Shelley et al. (2018b) for morphological appraisal. | *Morphological analysis* (46 characters), *Mitochondrial Marker*: cytb (1140 bp). *Nuclear Markers*: ~500 UCE loci. |
| *Hypseleotris regalis* II | J. Shelley, P. Unmack & C. Thacker (unpublished genetic data), J. Shelley (unpublished morphological data). Also see Shelley et al. (2018b) for morphological appraisal. | *Morphological analysis* (46 characters), *Mitochondrial Marker*: cytb (1140 bp). *Nuclear Markers*: ~500 UCE loci. |
| *Glossogobius* sp. 3 (dwarf) | M. hammer (unpublished genetic data), G. Allen (unpublished morphological data). Also see Allen et al. (2002) for morphological appraisal. | Morphology appraisal, *Mitochondrial Marker*: cytb (1140 bp). |
| **References**  Allen G.R., Midgley S.H., & Allen M. (2002) *Field Guide to the Freshwater Fishes of Australia.* Western Australian Museum, Western Australia, Perth.  Cook B.D., Adams M., Unmack P.J., Burrows D., Pusey B.J., Perna C., & Hughes J.M. (2017) Phylogeography of the mouth-brooding freshwater fish *Glossamia aprion* (Apogonidae) in northern and eastern Australia: historical biogeography and allopatric speciation. *Biological Journal of the Linnean Society*, blx035.  Cook B.D., Unmack P.J., Huey J. a., & Hughes J.M. (2014) Did common disjunct populations of freshwater fishes in northern Australia form from the same biogeographic events? *Freshwater Science*, **33**, 263–272.  Hogan, A.E. & Vallance, T.D. (2005) Rapid assessment of fish biodiversity in southern Gulf of Carpentaria catchments. Project Report Number QI04074, Queensland. Department of Primary Industries and Fisheries, Walkamin, 148 pp.  Huey J. A, Cook B.D., Unmack P.J., & Hughes J.M. (2014) Broadscale phylogeographic structure of five freshwater fishes across the Australian Monsoonal Tropics. *Bioone*, **33**, 273–287.  McGlashan D.J. & Hughes J.M. (2001) Low levels of genetic differentiation among populations of the freshwater fish *Hypseleotris compressa* (Gobiidae: Eleotridinae): implications for its biology, population connectivity and history. *Heredity*, **86**, 222–233.  Shelley J.J., Morgan D.. L., Hammer M.P., C. L.F.M., Moore G.I., Gomon M.F., Allen M.G., & Saunders T. (2018a) *A field guide to the freshwater fishes of the Kimberley.* Murdoch University Print Production Team, Perth.  Shelley J.J., Swearer S.E., Adams M., Dempster T., Le Feuvre M.C., Hammer M.P., & Unmack P.J. (2018b) Cryptic biodiversity in the freshwater fishes of the Kimberley endemism hotspot, northwestern Australia. *Molecular Phylogenetics and Evolution*, **127**, 843–858.  Unmack P.J., Allen G.R., & Johnson J.B. (2013) Phylogeny and biogeography of rainbowfishes (Melanotaeniidae) from Australia and New Guinea. *Molecular Phylogenetics and Evolution*, **67**, 15–27.  Unmack P.J. & Dowling T.E. (2010) Biogeography of the genus *Craterocephalus* (Teleostei: Atherinidae) in Australia. *Molecular Phylogenetics and Evolution*, **55**, 968–984.  Wong B.B.M., Keogh J.S., & McGlashan D.J. (2004) Current and historical patterns of drainage connectivity in eastern Australia inferred from population genetic structuring in a widespread freshwater fish *Pseudomugil signifer* (Pseudomugilidae). *Molecular Ecology*, **13**, 391–401.  **Lead authors of unpublished data**  Peter J. Unmack: Institute for Applied Ecology, University of Canberra, ACT 2601, Australia  Michael P. Hammer: Natural Sciences, Museum and Art Gallery of the Northern Territory, PO Box 4646, Darwin, NT 0801, Australia  James J. Shelley & Matthew C. Le Feuvre: School of BioSciences, University of Melbourne, Vic 3010, Australia  Mark Adams: Evolutionary Biology Unit, South Australian Museum, North Terrace, SA 5000, Australia  Christine E. Thacker: Vertebrates-Ichthyology, Natural History Museum of Los Angeles County, 900 Exposition Blvd., Los Angeles, CA 90007, USA  David L. Morgan: Freshwater Fish Group & Fish Health Unit, Centre for Fish & Fisheries Research, Murdoch University, Western Australia, 6150, Australia | | |

Gene codes: 18S ribosomal RNA (18S), ATPase 6 (ATP6), ATPase 8 (ATP8), cytochrome oxidase subunit III (COIII), cytochrome b (cytb), mitochondrially encoded NADH: ubiquinone oxidoreductase core subunit 4L (ND4L), mitochondrially encoded NADH: ubiquinone oxidoreductase core subunit 4 (ND4), mitochondrially encoded NADH: ubiquinone oxidoreductase core subunit 5 (ND5), mitochondrially encoded NADH: ubiquinone oxidoreductase core subunit 6 (ND6), nuclear recombination activation gene one (RAG1), nuclear S7 ribosomal protein gene (S7), single nuclear polymorphism (SNP), ultra-conserved element (UCE).

Appendix 3

| **Family / Species (candidates and described)** | **Province** | **Subprovince** |
| --- | --- | --- |
| **Osteoglossidae** |  |  |
| *Scleropages jardinii* | Northern, Eastern | Top End, Northern Gulf, Southern Gulf, Cape York Peninsula |
| **Anguillidae** |  |  |
| *Anguilla bicolor* | Kimberley, Northern | Southern lowlands, West Plateau, NW Plateau, North Plateau, Eastern Lowlands, Daly, Top End |
| *Anguilla obscura* | Eastern | Cape York Peninsula, Lakefield, Wet Tropics, Burdekin |
| *Anguilla marmorata* | Eastern | Wet Tropics |
| *Anguilla reinhardtii* | Eastern | Cape York Peninsula, Lakefield, Wet Tropics, Burdekin |
| **Muraenidae** |  |  |
| *Gymnothorax polyuranodon* | Eastern | Lakefield, Wet Tropics |
| **Clupeidae** |  |  |
| *Clupeoides cf. papuensis* | Northern | Southern Gulf |
| *Nematalosa erebi* I (outside of the Pilbara) | Kimberley, Northern, Eastern | Southern lowlands, West Plateau, NW Plateau, North Plateau, Eastern Lowlands, Victoria Basin, Daly, Top End, Northern Gulf, Southern Gulf, Cape York Peninsula, Lakefield, Wet Tropics, Burdekin |
| **Engraulidae** |  |  |
| *Thryssa scratchleyi* | Kimberley, Northern | Southern Lowlands, Top End, Northern Gulf, Southern Gulf |
| **Ariidae** |  |  |
| *Cinetodus froggatti* | Northern | Top End, Southern Gulf |
| *Hemiarius dioctes* | Northern | Top End, Southern Gulf |
| *Neoarius berneyi* | Northern | Daly, Top End, Northern Gulf, Southern Gulf |
| *Neoarius graeffei* | Kimberley, Northern, Eastern | Southern lowlands, NW Plateau, North Plateau, Eastern Lowlands, Victoria Basin, Daly, Top End, Northern Gulf, Southern Gulf, Cape York Peninsula, Burdekin |
| *Neoarius leptaspis* | Kimberley, Northern, Eastern | Eastern Lowlands, Victoria Basin, Daly, Top End, Southern Gulf, Northern Gulf, Cape York Peninsula |
| *Neoarius midgelyorum* | Kimberley, Northern | Southern lowlands, West Plateau, NW Plateau, North Plateau, Eastern Lowlands, Victoria Basin, Daly, Top End |
| *Neoarius paucus* | Northern, Eastern | Southern Gulf, Northern Gulf, Cape York Peninsula, Lakefield |
| **Plotosidae** |  |  |
| *Anodontiglanis dahli* | Kimberley, Northern | Southern Lowlands, Victoria Basin, Daly, Top End, Northern Gulf, Southern Gulf |
| *Neosilurus ater* | Kimberley, Northern, Eastern | Southern lowlands, West Plateau, NW Plateau, North Plateau, Eastern Lowlands, Victoria Basin, Daly, Top End, Northern Gulf, Southern Gulf, Cape York Peninsula, Lakefield, Wet Tropics, Burdekin |
| *Neosilurus brevidorsalis* | Northern, Eastern | Northern Gulf, Cape York Peninsula |
| *Neosilurus hyrtlii* I (east of Cato R.) | Northern, Eastern | Northern Gulf, Southern Gulf, Cape York Peninsula, Lakefield, Wet Tropics, Burdekin |
| *Neosilurus hyrtlii* II (Durack R. - Cato R.) | Kimberley, Northern | Eastern Lowlands, Victoria Basin, Daly, Top End |
| *Neosilurus hyrtlii* III (Fitzroy R. - King George R.) | Kimberley | Southern lowlands, West Plateau, NW Plateau, North Plateau |
| *Neosilurus mollespiculum* | Eastern | Burdekin |
| *Neosilurus pseudospinosus* I (Durack R. - Finniss R.) | Kimberley, Northern | Eastern Lowlands, Victoria Basin, Daly |
| *Neosilurus pseudospinosus* II (Fitzroy R. - Drysdale R.) | Kimberley | Southern lowlands, West Plateau, NW Plateau, North Plateau |
| *Porochilus argenteus* | Northern | Southern Gulf |
| *Porochilus obbesi* | Northern, Eastern | Daly, Top End, Northern Gulf, Cape York Peninsula, Lakefield |
| *Porochilus rendahli* | Kimberley, Northern, Eastern | Southern Lowlands, North Plateau, Eastern Lowlands, Victoria Basin, Daly, Top End, Northern Gulf, Southern Gulf, Cape York Peninsula, Lakefield, Wet Tropics, Burdekin |
| *Tandanus tropicanus* | Eastern | Wet Tropics |
| **Zenarchopteridae** |  |  |
| *Zenarchopterus* spp. | Northern | Top End, Northern Gulf, Southern Gulf |
| **Hemiramphidae** |  |  |
| *Arrhamphus sclerolepis* | Kimberley, Northern, Eastern | West Plateau, NW Plateau, Eastern Lowlands, Victoria Basin, Daly, Top End, Northern Gulf, Southern Gulf, Cape York Peninsula, Lakefield, Wet Tropics, Burdekin |
| **Belonidae** |  |  |
| *Strongylura krefftii* | Kimberley, Northern, Eastern | Southern lowlands, NW Plateau, North Plateau, Eastern Lowlands, Victoria Basin, Daly, Top End, Northern Gulf, Southern Gulf, Lakefield, Wet Tropics, Burdekin |
| **Atherinidae** |  |  |
| *Craterocephalus helenae* | Kimberley | North Plateau |
| *Craterocephalus marianae* | Northern | Daly, Top End |
| *Craterocephalus munroi* | Northern | Southern Gulf |
| *Craterocephalus lentiginosus* I (King Ed R. - Goyder R.) | Kimberley, Northern | North Plateau, Victoria Basin, Daly, Top End |
| *Craterocephalus lentiginosus* II (Fitzroy R.) | Kimberley | Southern Lowlands |
| *Craterocephalus lentiginosus* III (Calder R. - Prince Regent R.) | Kimberley | West Plateau, NW Plateau |
| *Craterocephalus stercusmuscarum* I (Buckingham R. - Johnstone R.) | Northern, Eastern | Top End, Northern Gulf, Southern Gulf, Cape York Peninsula, Lakefield, Wet Tropics |
| *Craterocephalus stercusmuscarum* II (Johnstone R. and Herbert R.) | Eastern | Wet Tropics, Burdekin |
| *Craterocephalus stercusmuscarum* III (Johnstone R. - O'Connell R.) | Eastern | Wet Tropics, Burdekin |
| *Craterocephalus stramineus* I (Daly R. - Reynolds R.) | Northern | Daly |
| *Craterocephalus stramineus* II (Durack R. - Victoria R.) | Kimberley | Eastern lowlands, Victoria Basin |
| *Craterocephalus stramineus* III (Nicholson R. - Leichhardt R.) | Northern | Southern Gulf |
| **Melanotaeniidae** |  |  |
| *Cairnsichthys rhombosomoides* | Eastern | Wet Tropics |
| *Cairnsichthys bitaeniatus* | Eastern | Wet Tropics |
| *Iriatherina werneri* I (Coelman R. - Olive R.) | Northern, Eastern | Southern Gulf, Northern Gulf, Cape York Peninsula |
| *Iriatherina werneri* II (Liverpool R. - Goyder R.) | Northern | Top End |
| *Melanotaenia australis* | Kimberley, Northern | Southern lowlands, West Plateau, NW Plateau, North Plateau, Eastern Lowlands, Victoria Basin, Daly, Top End |
| *Melanotaenia eachamensis* | Eastern | Wet Tropics |
| *Melanotaenia exquisita* I (Daly R. - South Alligator R.) | Northern | Daly, Top End |
| *Melanotaenia exquisita* II (King George R. - Fitzmaurice R.) | Kimberley | North Plateau, Eastern lowlands, Victoria Basin |
| *Melanotaenia gracilis* | Kimberley | North Plateau |
| *Melanotaenia maccullochi* I (Jardine R. – Endeavour R.) | Eastern | Cape York Peninsula, Lakefield |
| *Melanotaenia maccullochi* II (Reynolds R. - Finnis R.) | Northern | Daly |
| *Melanotaenia maccullochi* III (Daintree R. - Murray R. (QLD)) | Eastern | Wet Tropics, Burdekin |
| *Melanotaenia nigrans* | Kimberley, Northern, Eastern | North Plateau, Victoria Basin, Daly, Top End, Northern Gulf, Southern Gulf, Cape York Peninsula |
| *Melanotaenia pygmaea* | Kimberley | NW Plateau |
| *Melanotaenia splendida* | Northern, Eastern | Top End, Northern Gulf, Southern Gulf, Cape York Peninsula, Lakefield, Wet Tropics, Burdekin |
| *Melanotaenia trifasciata* I | Northern | Top End |
| *Melanotaenia trifasciata* II | Northern | Top End |
| *Melanotaenia trifasciata* III | Northern, Eastern | Top End, Northern Gulf, Cape York Peninsula, Lakefield |
| *Melanotaenia trifasciata* IV | Eastern | Wet Tropics |
| *Melanotaenia utcheensis* | Eastern | Wet Tropics |
| **Pseudomugilidae** |  |  |
| *Pseudomugil gertrudae* | Northern, Eastern | Daly, Top End, Northern Gulf, Cape York Peninsula, Lakefield, Wet Tropics, Burdekin |
| *Pseudomugil signifer* I (*P. signifier* II outside the AMT) | Northern, Eastern | Northern Gulf, Cape York Peninsula, Lakefield, Wet Tropics, Burdekin |
| *Pseudomugil tenellus* | Northern, Eastern | Daly, Top End, Northern Gulf, Southern Gulf, Cape York Peninsula, Lakefield |
| **Synbranchidae** |  |  |
| *Monopterus albus* | Eastern | Cape York Peninsula, Wet Tropics |
| *Ophisternon* spp. | Kimberley, Northern, Eastern | Victoria Basin, Daly, Top End, Northern Gulf, Southern Gulf, NE Cape, Lakefield, Wet Tropics, Burdekin |
| **Tetrarogidae** |  |  |
| *Notesthes robusta* | Eastern | Lakefield, Wet Tropics, Burdekin |
| **Latidae** |  |  |
| *Lates calcarifer* | Kimberley, Northern, Eastern | Southern lowlands, West Plateau, NW Plateau, North Plateau, Eastern Lowlands, Victoria Basin, Daly, Top End, Northern Gulf, Southern Gulf, Cape York Peninsula, Lakefield, Wet Tropics, Burdekin |
| **Ambassidae** |  |  |
| *Ambassis agassizii* | Eastern | Lakefield, Wet Tropics, Burdekin |
| *Ambassis agrammus* | Kimberley, Northern, Eastern | Victoria Basin, Daly, Top End, Northern Gulf, Southern Gulf, Cape York Peninsula, Lakefield, Wet Tropics, Burdekin |
| *Ambassis elongata* | Kimberley, Northern, Eastern | Southern Lowlands, Eastern Lowlands, Northern Gulf, Southern Gulf, Cape York Peninsula |
| *Ambassis macleayi* | Kimberley, Northern, Eastern | North Plateau, Eastern lowlands, Victoria Basin, Daly, Top End, Northern Gulf, Southern Gulf, Cape York Peninsula, Lakefield, Wet Tropics |
| *Ambassis miops* | Eastern | Lakefield, Wet Tropics, Burdekin |
| *Ambassis* sp. 1 “muelleri” | Kimberley, Northern, Eastern | Southern lowlands, West Plateau, NW Plateau, North Plateau, Eastern Lowlands, Victoria Basin, Daly, Top End, Northern Gulf, Southern Gulf, Lakefield, Burdekin |
| *Ambassis* sp. 2 | Kimberley | Southern Lowlands |
| *Denariusa bandata* | Northern, Eastern | Daly, Top End, Northern Gulf, Southern Gulf, Cape York Peninsula, Lakefield, Wet Tropics, Burdekin |
| *Parambassis gulliveri* | Kimberley, Northern | Eastern Lowlands, Victoria Basin, Daly, Southern Gulf |
| **Percicthyidae** |  |  |
| *Guyu wujalwujalensis* | Eastern | Wet Tropics |
| **Terapontidae** |  |  |
| *Amniataba* sp. 1 (Charnley R. - Glenelg R.) | Kimberley | West Plateau |
| *Amniataba percoides* I (Rivers outside of the Kimberley) | Northern, Eastern | Daly, Top End, Northern Gulf, Southern Gulf, Cape York Peninsula, Lakefield, Wet Tropics, Burdekin |
| *Amniataba percoides* II (Ord R. - Victoria R.) | Kimberley | Eastern Lowlands, Victoria Basin |
| *Amniataba percoides* III (Pentecost R. - Durack R.) | Kimberley | Eastern Lowlands |
| *Amniataba percoides* IV (King Edward R. - Drysdale R.) | Kimberley | North Plateau |
| *Amniataba percoides* V (Fitzroy R. - Isdell R.) | Kimberley | Southern Lowlands |
| *Hannia greenwayi* I (Fitzroy R. - Glenelg R.) | Kimberley | Southern Lowlands, West Plateau |
| *Hannia greenwayi* II (Prince Regent R. - Roe R.) | Kimberley | NW Plateau |
| *Hephaestus carbo* | Northern, Eastern | Top End, Northern Gulf, Southern Gulf, Cape York Peninsula |
| *Hephaestus epirrhinos* | Kimberley | North Plateau |
| *Hephaestus fuliginosus* | Northern, Eastern | Daly, Top End, Northern Gulf, Southern Gulf, Cape York Peninsula, Wet Tropics, Burdekin |
| *Hephaestus tulliensis* | Eastern | Wet Tropics |
| *Hephaestus jenkinsi* | Kimberley | Southern lowlands, West Plateau, NW Plateau, North Plateau, Eastern Lowlands, Victoria Basin |
| *Leiopotherapon macrolepis* | Kimberley | NW Plateau |
| *Leiopotherapon unicolor* | Kimberley, Northern, Eastern | Southern lowlands, West Plateau, NW Plateau, North Plateau, Eastern Lowlands, Victoria Basin, Daly, Top End, Northern Gulf, Southern Gulf, Cape York Peninsula, Lakefield, Wet Tropics, Burdekin |
| *Pingalla gilberti* | Northern | Southern Gulf |
| *Pingalla lorentzi* | Northern, Eastern | Daly, Northern Gulf, Cape York Peninsula |
| *Pingalla midgleyi* | Northern | Daly, Top End |
| *Scortum neili* | Kimberley | Victoria Basin |
| *Scortum ogilbyi* | Northern | Southern Gulf, Northern Gulf |
| *Scortum parviceps* | Eastern | Burdekin |
| *Syncomistes butleri* | Northern | Top End |
| *Syncomistes bonapartensis* | Kimberley, Northern | North Plateau, Eastern lowlands, Victoria Basin, Daly |
| *Syncomistes kimberleyensis* | Kimberley | Eastern lowlands |
| *Syncomistes rastellus* | Kimberley | North Plateau |
| *Syncomistes trigonicus* | Kimberley | NW Plateau, North Plateau |
| *Syncomistes wunambal* | Kimberley | North Plateau |
| *Syncomistes holsworthi* | Kimberley | Eastern Lowlands, Victoria Basin |
| *Syncomistes versicolor* | Kimberley | NW Plateau |
| *Syncomistes dilliensis* | Kimberley | West Plateau |
| *Syncomites moranensis* | Kimberley | NW Plateau |
| *Syncomites carcharus* | Kimberley | NW Plateau |
| *Variichthys lacustris* | Northern | Southern Gulf |
| **Kuhliidae** |  |  |
| *Kuhlia marginata* | Eastern | Cape York Peninsula, Wet Tropics |
| *Kuhlia rupestris* | Northern | Cape York Peninsula, Lakefield, Wet Tropics, Burdekin |
| **Apogonidae** |  |  |
| *Glossamia aprion* I (Ord R. and west) | Kimberley | Southern lowlands, West Plateau, NW Plateau, North Plateau, Eastern Lowlands |
| *Glossamia aprion* II (Victoria R. - Cato R.) | Kimberley, Northern | Victoria Basin, Daly, Top End |
| *Glossamia aprion* III (Giddy R. - Johnson R.) | Northern, Eastern | Northern Gulf, Southern Gulf, Cape York Peninsula, Lakefield, Wet Tropics |
| *Glossamia aprion* IV (Tully R. - Budekin R.) | Eastern | Wet Tropics, Burdekin |
| **Toxotidae** |  |  |
| *Toxotes chatareus* | Kimberley, Northern, Eastern | NW Plateau, North Plateau, Eastern Lowlands, Victoria Basin, Daly, Top End, Northern Gulf, Southern Gulf, Cape York Peninsula, Lakefield, Wet Tropics, Burdekin |
| *Toxotes lorentzi* | Northern | Daly, Top End |
| *Toxotes* *kimberleyensis* | Kimberley | Southern Lowlands, West Plateau |
| **Eleotridae** |  |  |
| *Bostrichthys zonatus* | Northern | Top End, Northern Gulf, Southern Gulf |
| *Bunaka gyrinoides* | Eastern | Cape York Peninsula, Lakefield, Wet Tropics, Burdekin |
| *Giurus margaritacea* | Northern, Eastern | Northern Gulf, Cape York Peninsula, Lakefield, Wet Tropics, Burdekin |
| *Hypseleotris barrawayi* | Northern | Daly |
| *Hypseleotris compressa* | Kimberley, Northern, Eastern | Southern lowlands, West Plateau, NW Plateau, North Plateau, Eastern Lowlands, Victoria Basin, Daly, Top End, Northern Gulf, Southern Gulf, Cape York Peninsula, Lakefield, Wet Tropics, Burdekin |
| *Hypseleotris klunzingeri* | Eastern | Burdekin |
| *Hypseleotris* sp. “midgley” | Eastern | Burdekin |
| *Hypseleotris ejuncida* I (Prince Regent R.) | Kimberley | NW Plateau |
| *Hypseleotris ejuncida* II (King Edward R.) | Kimberley | North Plateau |
| *Hypseleotris kimberleyensis* I (Fitzroy R.) | Kimberley | Southern Lowlands |
| *Hypseleotris kimberleyensis* II (Calder R. - Charnley R.) | Kimberley | West Plateau |
| *Hypseleotris regalis* I (Prince Regent R.) | Kimberley | NW Plateau |
| *Hypseleotris regalis* II (Roe) | Kimberley | NW Plateau |
| *Kimberleyeleotris hutchinsi* | Kimberley | North Plateau |
| *Kimberleyeleotris notata* | Kimberley | North Plateau |
| *Mogurnda adspersa* | Eastern | Lakefield, Wet Tropics, Burdekin |
| *Mogurnda mogurnda* | Kimberley, Northern, Eastern | Eastern Lowlands, Victoria Basin, Daly, Top End, Southern Gulf, Northern Gulf, Cape York Peninsula, Lakefield, Wet Tropics, Burdekin |
| *Mogurnda oligolepis* | Kimberley | Southern lowlands, West Plateau, NW Plateau, North Plateau, Eastern Lowlands |
| *Oxyeleotris aruensis* | Northern, Eastern | Top End, Northern Gulf, Cape York Peninsula, Wet Tropics, Burdekin |
| *Oxyeleotris fimbriata* | Northern, Eastern | Northern Gulf, Cape York Peninsula |
| *Oxyeleotris lineolata* | Kimberley, Northern, Eastern | North Plateau, Eastern lowlands, Victoria Basin, Daly, Top End, Northern Gulf, Southern Gulf, Cape York Peninsula, Lakefield, Wet Tropics, Burdekin |
| *Oxyeleotris selheimi* | Kimberley, Northern, Eastern | Southern lowlands, North Plateau, Eastern Lowlands, Victoria Basin, Daly, Top End, Northern Gulf, Southern Gulf, Cape York Peninsula, Lakefield, Wet Tropics |
| *Oxyeleotris nullipora* | Northern, Eastern | Daly, Top End, Northern Gulf, Southern Gulf, Cape York Peninsula, Lakefield, Burdekin |
| *Philypnodon grandiceps* | Eastern | Burdekin |
| **Gobiidae** |  |  |
| *Awaous acritosus* | Eastern | Cape York Peninsula, Lakefield, Wet Tropics, Burdekin |
| *Awaous ocellaris* | Eastern | Wet Tropics |
| *Chlamydogobius ranunculus* | Kimberley, Northern, Eastern | Eastern Lowlands, Victoria Basin, Top End, Southern Gulf, Northern Gulf, Cape York Peninsula, Burdekin |
| *Glossogobius aureus* | Kimberley, Northern, Eastern | Victoria Basin, Daly, Top End, Southern Gulf, Northern Gulf, Lakefield, Wet Tropics |
| *Glossogobius bellendenensis* | Eastern | Wet Tropics |
| *Glossogobius concavifrons* | Northern, Eastern | Top End, Northern Gulf, Cape York Peninsula |
| *Glossogobius giuris* | Kimberley, Northern, Eastern | Southern lowlands, West Plateau, NW Plateau, North Plateau, Eastern Lowlands, Victoria Basin, Daly, Top End, Northern Gulf, Southern Gulf, Cape York Peninsula, Lakefield, Wet Tropics, Burdekin |
| *Glossogobius illimis* | Eastern | Cape York Peninsula, Lakefield, Wet Tropics, Burdekin |
| *Glossogobius* sp. “dwarf” | Northern, Eastern | Top End, Northern Gulf, Cape York Peninsula, Lakefield |
| *Glossogobius munroi* | Kimberley, Northern, Eastern | NW Plateau, Eastern Lowlands, Victoria Basin, Daly, Top End, Southern Gulf, Northern Gulf, Cape York Peninsula |
| *Mugilogobius notospilus* | Eastern | Lakefield, Wet Tropics, Burdekin |
| *Schismatogobius hoesei* | Eastern | Lakefield, Wet Tropics |
| *Sicyopterus lagocephalus* | Eastern | Wet Tropics |
| *Sicyopus discordipinnis* | Eastern | Wet Tropics |
| *Smilosicyopus fehlmanni* | Eastern | Wet Tropics |
| *Stenogobius psilosinionus* | Eastern | Wet Tropics |
| *Stiphodon atratus* | Eastern | Wet Tropics |
| *Stiphodon birdsong* | Eastern | Wet Tropics |
| *Stiphodon rutilaureus* | Eastern | Wet Tropics |
| *Stiphodon semoni* | Eastern | Wet Tropics |
| **Kurtidae** |  |  |
| *Kurtus gulliveri* | Kimberley, Northern | Eastern Lowlands, Victoria Basin, Daly, Top End, Southern Gulf |
| **Cynoglossidae** |  |  |
| *Cynoglossus heterolepis* | Northern | Daly, Top End |
| **Soleidae** |  |  |
| *Brachirus salinarum* | Northern | Northern Gulf, Southern Gulf |
| *Brachirus selheimi* | Northern | Top End, Northern Gulf, Southern Gulf |
| *Synclidopus hogani* | Eastern | Wet Tropics |
| *Leptachirus* spp. | Northern | Top End |
| *Leptachirus triramus* | Kimberley, Northern | Eastern Lowlands, Victoria Basin, Daly |
